# Supplementary material for: Herb–Drug Interactions: Worlds Intersect with the Patient at the Center
Source: Medicines (Basel). 2021 Aug 5;8(8):44. doi: 10.3390/medicines8080044 (PMC8401017; doi:10.3390/medicines8080044)
Supplement: Supplementary file 1 [file medicines-08-00044-s001.zip › medicines-1300928 supp proofdone.pdf]

Supplementary Materials: Herb–Drug Interactions: Worlds Intersect with the Patient at the Center

Mary Beth Babos, Michelle Heinan, Linda Redmond, Fareeha Moiz, Joao Victor Souza-Peres, Valerie Samuels, Tarun Masimukku, David Hamilton, Myra Khalid and Paul Herscu

Table S1. Pharmacokinetic pathways for drugs involved in cases matched to herbs in clinical studies. See text for details. Reference [36] unless specified otherwise. S substrate, major pathway; s substrate, minor pathway; X inhibitor, strong; x inhibitor, weak; I inducer, strong; i inducer, weak; *multiple isoforms.

	1A2	2A6	2B6	2C8/9	2C19	2D6	2E1	3A4/5	UGT*	BCRP/SEP	OAT*	2-Oct	PGP
alprazolam								S.x					
buspirone						s,x		S					
clozapine	S	s		s	s	s		S					
cyclosporine				x				S, x		x	x		S,x
dolutegravir								s	s	S		X	s
efavirenz				s	S			s					
efavirenz			S,I		i		S						
erlotinib	S		S					S,x					
estradiol	s, I		s	s	s	s		S					
etoposide	s						s	S					S
fluindione				S				S					
fluoxetine	s		s	s	s, X	s, X	s	s					
gefitinib						s		S		S			
Ibuprofen [74]				s	s, X			s	s		X		
imatinib	s				s	s		S,I					s
lamotrigine [37]									S			X	
lansoprazole				s	S			s					
lorazepam [37]									S				
norethindrone								S					
paclitaxel				S				s					
paroxetine								S					
phenelzine													

phenytoin	i		i	S	S			s, I	I				I
raltegravir									S				
rosuvastatin				s				s		S	S		
sertraline			s	s	s	s							
simvastatin								S			S		
tacrolimus								S					S
theophylline	S						s	s					
trazodone						s		S					
valproate [37]		s	s		s		s		S				
venlafaxine				s	s	s,x		s					
warfarin						S, X		s					

Table S2. Findings of clinical trials by herb and reference. ADE adverse drug effect; AG/I AUC glucose and insulin, B bleeding time, C clotting time, Chol cholesterol, Pl platelet aggregation, PS pupil size, 1B1 OATP1B1, PK pharmacokinetics, PD pharmacodynamics.

	1A2	2C8/9	2C19	2D6	2E1	3A4	NAT	XO	UGT	PGP	1B1	PK	PD
**Reference**	*Actea racemosa*												
Gurley [75]	0			+	0	0							
Gurley [76]										0			
	Allium sativum												
Abdul [77]													
Gurley [78]	0			0	+	0						0	
Gurley [79]	0			0	+	0							
	Astragalus spp												
Zhou [80]										0			
	**Baicalin**												
Fan [81]											+		
	Citrus paradisi												
Hu [82]											+		
	Corolus versicolor												
Nicandro [83]						0							
	Crataegus oxycantha												
Tankanow [84]										0			
Walker [85]													ADE

						Curcuma longa							
Al-Janoobi [20]				+		0							
Ikehata [86]						0						0	
						**EGCG**							
Kim [87]											+		
						Echinacea purpura							
Molto [88]						**0**							
						Eurycoma longifolia							
Salman [89]												**+**	
						**Genistein**							
Xiao [90]						**+**				**+**			
						Ginkgo biloba							
Aruna [91]													B
Aruna [91]													Pl
Dai [92]													Chol
Fan [93]										-			
Guo [94]						-				-			
Gurley [78]	0			0	0	0							
Gurley [79]	0			0	0	0							
Jiang [95]		0										0	
Kim [96]			0			0							Pl, B
Mohutsky [97]		0											
	**1A2**	**2C8/9**	**2C19**	**2D6**	**2E1**	**3A4**	**NAT**	**XO**	**UGT**	**PGP**	**1B1**	**PK**	**PD**
Zadoyan [98]	0	0	0	0		0							
						Hibiscus sabdariffa							
Souriti [54]												B	
						Humulus lupulus							
Vanbreeman [99]	0	0		0		0							
						Hydrastis canadensis							
Gurley [75]	0			+	0	+							
Gurley [100]										0			
Gurley [101]						+							
						Hypericum perforatum							

Andrén [102]		`											-
Bell [103]						0				0			
Bell]104]		0											
Nieminen [105]												-	-
Fan [106]		0											
Goey [107]						-							
Gurley [78]	0			0	-	-							
Gurley [79]	0			0	-	-							
Hennessy [108]										-			
Jiang [109]		-				-							
Jiang [95]		-										-	
Loughren [110]												0	PS
Markert [111]			-										
Mueller [112]						-							
Mueller [113]						-							
Murphy [114]													
Piscitelli [115]						-						-	
Portoles [116]						-							
Xie [117]						-				-			
Xu [118]												-	AG/I
	***Lavendula* as Silexan**												
Heger [119]													
	**Multiherbal**												
Chen [120]			0	0		0							
Fan [121]		-				-							
Kim [122]				0									
Nakayo [123]	0	0		0		0	0	0					
Park [124]												-	
Saruwatri [125]	+			0		0	0	0					
	**1A2**	**2C8/9**	**2C19**	**2D6**	**2E1**	**3A4/5**	**NAT**	**XO**	**UGT**	**PGP**	**1B1**	**PK**	**PD**
Wang [126]												0	INR
	Panax ginseng												
Gurley [77]	0			0	0	0							

Gurley [75]	0			+	0	0					
Jiang [109]		0				0					
Jiang [95]		-								-	
Lee [127]											INR
Malati [128]						-		0			
Phyllanthus emblica											
Fatima [129]											C, B
Piper methysticum											
Gurley [77]	0			0	+	0					
Gurley [78]								0			
Gurley [100]						0					
**Resveretrol**											
Bedada [130]					+						
Schisandra											
Jiang [109]										+	
Silybum marianum											
Fuhr [131]						0					
Gurley [76]								0			
Suzuki [132]	0	0		0		0					
Undaria pinnatifida											
Tocaciu [133]										0	
Vaccinium spp.											
Abdul [77]										0	
Valeriana officinale											
Gurley [74]	0			0	0	0					
Viscum alba											
Thronicke [134]											ADE
Zingiber officinale											
Jiang [95]		0								0	

- increased drug effect
- decreased drug effect
- benefit
- not studied
- no interaction

Table S3. Heat map for cases and interaction checkers, X "Increased INR meaning decreased efficacy"; **A** = Memorial Sloan Kettering Cancer Center [40] **B** = Medscape [39] **C** = Natural Medicines Database [41] **D** = Lexicomp Drug Interactions [36] **E** = Integrative Pro [38] **F** = Stockley's Herb Drug Interaction [16]. *NOS specific species of ginseng not reported in case; warfarin was a proxy for less common vitamin K antagonists .

Botanical	Drug name	Cases	A	B	C	D	E	F
Allium sativum multiherb	lansoprazole	-1	0	0	-1	0	0	0
Aloe barbadensis	sevoflurane	1	1	0	1	0	0	1
Angelica sinensis	warfarin	1	1	1	1	1	1	1
A. graveolens multiherb	venlafaxine	1		0	1	1	0	0
Areca catechu	cyclophosphamide	1		0	0	0		0
Areca catechu	doxorubicin	1		0	1	0		0
Areca catechu	paclitaxel	1		0	0	0		0
Aronia melanocarpa	trabectedin	1			1		0	0
Artemisia absinthum	warfarin	1	0		-1		0	0
Bee pollen	warfarin	1	1	0	1	1	0	0
Berberine	tacrolimus	1	1	0	1	1	0	0
Camellia sinensis	warfarin	1	0	-1	1	1	0	-1
Camellia sinensis	simvastatin	1	0	0	1	1	0	0
Cathus edulus	bupivacaine	1		0	0			
Cathus edulus	sevoflurane	1		0	0		0	
Cathus edulus	propofol	1		0	0		0	
Cathus edulus	fentanyl	1		0	0		0	
Cathus edulus	anesthesia	1		0	0			
Commiphora molol	warfarin	-1	-1	0	-1			0
Curcuma longa	paclitaxel	1	1	0	1	0	0	0
Curcuma longa	tacrolimus	1	1	0	1	0	0	0
Curcuma longa	fluindione	1	0	0	0	0		0
Curcuma longa	warfarin proxy	1	1	0	1	1	0	0
Cynara scolymus	colchicine	1			0	1	0	0
Diosmin	trabectedin	1			1	0	0	0
Echinacea spp	etoposide	1	1	0	1	1	B	0
Echinacea spp, multiherb	lansoprazole	-1	0	0	-1	0	0	0
Eleuthrococcus senticosus	digoxin.	1	1	1	1	0	1	1
E. senticosus *NOS	lamotrigine	1		0	0	0	0	0

Equisetum arvense	efavirenz	-1		0	-1	0	0	0
Equisetum arvense	emtracitabine	-1			-1	0	0	0
Equisetum arvense	tenofovir	-1		0	-1	0	0	0
Equisetum arvense	zidovudine	-1		0	-1	0	0	0
Fish oil	warfarin	1	1	1	1	1	0	
F fomentarius multiherb	gefitinib	-1						
Ginkgo biloba	efavirenz	-2	-1	0	-1	-1	0	0
Ginkgo biloba	trazodone	1	1	0	1	0	1	1
Ginkgo biloba	aescinate	1	0	0	0			0
Ginkgo biloba	phenytoin	-1	0	0	-1	-1	0	-1
Ginkgo biloba	valproate	-1	0	0	-1	0	0	-1
Ginkgo biloba	ibuprofen	1	1	1	1	1	1	1
Ginkgo biloba w Hypericum	buspirone	1	0	0	-1	0	0	1
Ginkgo biloba w Hypericum	fluoxetine	1	0	0	-1	1	B	0
Ginseng NOS multiherb	lansoprazole	-1	0	0	0	0	0	0
Glucosamine chondroitin	warfarin	1	1	0	1	1	X	1
Glycine max	warfarin	-1	0	0	-1	0	0	-1
Glycyrrhiza glabra	enalapril	1	0	0	-1	-1	0	-1
Grifola frondosa	warfarin	1	1	0	1	1	1	
Hibiscus spp	erlotinib	1		0	0	1	0	
Hypericum multiherb	lansoprazole	-1	0	-1	-1	-1	0	-1
Hypericum perforatum	cyclosporin	1	-1	-1	-1	-1	-1	-1
Hypericum perforatum	sertraline	1	1	1	1	1	1	1
Hypericum perforatum	buspirone	1	0	1	1	1	0	1
Hypericum perforatum	estradiol	-1	-1	-1	-1	-1	0	0
Hypericum perforatum	oral contracept	-1	-1	0	-1	-1	-1	-1
Hypericum perforatum	tacrolimus	-1	-1	-1	-1	-1	0	-1
Hypericum perforatum	rosuvastatin	-1	-1	0	-1	0	0	-1
Hypericum perforatum	theophylline	-1	0	-1	-1	0	-1	-1
Hypericum perforatum	clozapine	-1	-1	-1	-1	0	0	0
Hypericum perforatum	venlafaxine	1	0	1	1	1	1	1
Hypericum multiherb	dolutegravir	0	-1	-1	-1	-1	0	0
Hypericum w Ginkgo	fluoxetine	1	1	1	1	1	0	1

Inonotus multiherb	geftinib	-1	0		0			
Liriope multiherb	warfarin	1						
Lycium barbarum	warfarin	1	1	0	1		0	1
Matricaria spp multiherb	warfarin	1	1	0	1	1	0	1
Matricaria spp multiherb	cyclosporin	-1	0	0	0		0	0
Momordica charantia	chlorpropamide	1	1	1	1	1		
Morinda citrifolia	warfarin	-1	-1	0	-1		0	
Morinda citrifolia	phenytoin	-1	-1	0	1		0	
Mitragyna speciosa	quetiapine	1	0	0	1	0		
Nattokinase	aspirin	1	1	0	1	0	0	1
Opuntia spp	glipizide	1		1	1	0	0	
Opuntia spp	metformin	1		1	1	0	0	
Panax ginseng multiherb	warfarin	1	-1	1	1	1	-1	1
Panax ginseng	imatinib	1	1	0	1	1	1	0
Panax ginseng	raltegravir	1	1	0	1	0	0	0
Panax ginseng	warfarin	-2	-1	1	-1	1	-1	-1
Panax ginseng	phenelzine	1	1	1	1	0	0	1
Panax ginseng, germanium	furosemide	-1	0	0	-1	0		0
Panax ginseng *NOS	lamotrigine		0	0	0	0	0	0
Panax NOS	lamotrigine	1		0	0	0	0	0
P. quinq. *NOS multiherb	geftinib	-1	0	0		0		
P. ginseng *NOS multiherb	geftinib	-1	0	0	-1	0		
*Eleuthero**NOS multiherb	geftinib	-1	0	0	-1	0		
Panax quinquefolius *NOS	lamotrigine	1		0	0	0		0
P. incarnata multiherb	lorazepam	1	0	0	1	0	0	0
P. incarnata multiherb	dolutegravir	0	0	0	0	0	0	0
Peumus boldos	tacrolimus	-1	0	0	-1	0	0	0
Peumus boldos	warfarin	1	1	0	1	0	0	1
Phellinus multiherb	geftinib	-1			0			
Piper methysticum	paroxetine	1	0	0	-1	1	0	
P methysticum multiherb	alprazolam	1	1	0	1	1	1	
Rhodiola rosa	escitalopram	1		0	0	1	0	0
Royal jelly	warfarin	1	1	0	1		0	

S. miltiorrhiza multiherb	warfarin	1	0	0	1		1	1
S. mlltiorrhiza multiherb	salicylate	1		1	0		0	1
Schizandra spp multiherb	warfarin	1	0	1	-1	0	0	-1
Stevia rebaudiana	simvastatin	1	0	0	0	0	0	
T. parthenium multiherb	lansoprazole	-1	0	0	1	0	0	0
T pratense or Meliotus	interferon 1b	1	0	0	0	0		0
T pratens or Meliotus	methotrexate	1	1	0	1	0	0	0
T foenum-gracum	warfarin	1	1	0	1	1	0	1
Uncaria tomentosa	azatanavir	1	1	0				1
Uncaria tomentosa	ritonavir	1	1	0	1	1	0	1
Uncaria tomentosa	saquinavir	1	1	0	1	1		1
Vaccinium spp	warfarin	1	1	1	1	1	1	1
V. officinalis, multiherb	paroxetine	1	0	1	1	1	0	0
V. officinalis, multiherb	lorazepam	1	1	1	1	1	0	1
V. officinalis, multiherb	dolutegravir	0	0	0	-1	0	0	0
Zingiber officinalis	phenprocoumon	1	1		1	1		1
Zingiber officinalis	warfarin proxy		1	1	1	1	0	1

increased effect target drug
dereased effect target drug
no interaction
benefit
no data

Table S4. Case reported drug interacting pairs with severity, reaction, and reliability index (RI) as a measure of report completeness. *RI = (number of items possible – number absent)/ total possible; see text for further detail. CK Creatine kinase, INR international normalized ratio, NMS neuroleptic malignant syndrome, AMI acute myocardial infarction, DRESS drug reaction with eosinophilia and systemic symptoms, ADE adverse drug event, NOS not otherwise specified. [a] Multiple cases, case series reporting.

Document	Ref.	Latin name	Drug	Severity	Reaction	RI*
Acikgoz 2013	[135]	*Artemisia absinthimum*	warfarin	severe	GI bleeding	0.80
Almeida 1996	[136]	*Piper methysticum*	alprazolam	minor	fever, headache, confusion	0.70
Alscher 2003	[137]	*H. perforatum* multiherbal	cyclosporine	minor	decreased level	0.60
Aslam 1979	[138]	*Momordica charantia*	chlorpropamide	minor	hypoglycemia	0.60
Barbanel 2000	[139]	*Hypericum perforatum*	sertraline	severe	mania	0.70
Barone 2000	[140]	*Hypericum perforatum*	cyclosprine	severe	decreased level	0.50
Becker 1996	[8]	*Panax ginseng, contaminant*	furosemide	severe	decreased efficacy	0.73

Document	Ref.	Latin name	Drug	Severity	Reaction	RI*
Bilgi 2010	[141]	*Panax ginseng*	imatinib	severe	hepatotoxicity	0.70
Bolley 2002	[142]	*Hypericum perforatum*	tacrolimus	minor	reduced level	0.90
Bossear 2012	[143]	*Echinacea spp* multiherb	etoposide	severe	thrombocytopenia	0.73
Breidenbach 2000	[144]	*Hypericum perforatum*	cyclosprine	minor	decreased level	0.80
Buckley 2005	[145]	Fish oil	warfarin	minor	additive	0.60
Bamgbade 2003 1[a]	[146]	*Cathus edulus*	general anesthesia	mild	additive	0.80
Bamgbade 2003 2[a]	[146]	*Cathus edulus*	general anesthesia	mild	additive	0.30
Bamgbade 2003 3[a]	[146]	*Cathus edulus*	bupivacaine	mild	additive	0.80
Campos 2018	[147]	*Cynara scolymus*	colchicine	severe	blood, liver, muscle toxicity	0.90
Cappuzzo 2006	[148]	multiherb	multidrug	mild	dizziness, hypotension	0.70
Carbajal 2014	[149]	*Peumus boldus*	tacrolimus	minor	decreased level	0.90
Carr 2004	[150]	*Morinda citrifolia*	warfarin	severe	decreased INR	0.90
Carrasco 2009	[151]	*P. incarnata, V. officinalis*	lorazepam	mild	movement disorder	0.80
Carter 2014	[152]	multiple herbs	busulfan	severe	increased level	0.80
Catteneo 2019	[153]	*H. perforatum, V. officinalis*	dolutegravir	none	no interaction	0.70
Chan 2019	[154]	*Stevia rebaudiana*	simvastatin	severe	myglobinuria, increased CK	0.64
Chang 2008	[155]	nattokinase	aspirin	severe	bleeding	0.80
Constable 2007	[156]	multiherb	lansoprazole	severe	GI bleeding	0.70
Cordova 2017 1[a]	[157]	*Equisetum arvense*	efavirenz, lamivudine, zidovudine	minor	increased viral load	0.50
Cordova 2017 2[a]	]157]	*Equisetum arvense*	efavirenz, emtracitabine, tenofovir	minor	increased viral load	0.60
Costa 2018	[9]	*C. longa* (contaminated)	paclitaxel	severe	hepatotoxicity	0.90
Damato 2017	[158]	Diosmin	trabectedin	severe	rhabdomyolysis	0.80
Dannawi 2002	[159]	*Hypericum perforatum*	buspirone	mild	serotonin syndrome	0.60
Daveluy 2014	[160]	*Curcuma longa*	fluindione	minor	increased INR	0.70
Epstein 2006	[161]	*Areca catechu*	cyclophosphamide, doxorubicin. paclitaxel	severe	mucositis	0.70
Al- Faraj 2005	[63]	*Commiphora molol*	warfarin	severe	increased INR	0.60

Document	Ref.	Latin name	Drug	Severity	Reaction	RI*
Galera 2008	[162]	*Uncaria tomentosa*	azatanavir, ritonavir, saquinavir	mild	increased level	0.70
Galluzzi 2000	[163]	*Ginkgo biloba*	trazodone	severe	coma	0.90
George 2011	[164]	*Vaccinium*	warfarin	minor	increased INR	0.50
Gordon 2009	[165]	*Hypericum perforatum*	rosuvastatin	minor	reduced efficacy	0.70
Griffiths 2008	[166]	*Vaccinium*	warfarin	fatal	bleeding, increased INR	0.90
Hanselin 2009	[167]	*Grifola frondosa*	warfarin	minor	increased INR	0.80
Hou 2013	[168]	berberine	tacrolimus	minor	increased level	0.91
Hughes 2018	[169]	*Mitragyna speciosa*	quetiapine	fatal	increased level, NMS	0.50
Hurren 2010	[170]	bee pollen	warfarin	minor	increased INR	0.40
Hwang 2008	[171]	multiherb	geftinib	mild	treatment failure	0.80
Iida 2006	[172]	*Glycyrrhiza glabra*	enalapril	severe	alkalosis and hypokalemia	0.80
Janetzky 1997	[173]	*Panax ginseng, ginseng*	warfarin	minor	decreased INR	0.91
Ji 2017	[174]	*Ginkgo biloba*	aescinate	severe	acute kidney injury	0.90
Jones 1987	[175]	*Panax ginseng*	phenelzine	mild	mania	0.60
Kang 2015	[176]	*Morinda citrifolia, noni*	phenytoin	severe	decreased level	0.82
Karhova 2000	[177]	*Hypericum perforatum*	cyclosporinee	severe	decreased level	0.80
Khalid 2016	[62]	*Apium graveolens, Hypericum*	venlafaxine	severe	mania, increased levels	0.80
Kiely 2002	[178]	*Glycine max, soy*	warfarin	minor	decreased INR	0.90
Kruth 2004	[179]	*Zingiber officinalis*	phenprocoumon	severe	bleeding	0.50
Kupiec 2005	[11]	*Ginkgo biloba* multiherb	divalproate, phenytoin	fatal	seizures	0.90
Lam 2001	[180]	*Lycium barbarum*	warfarin	minor	increased INR	1.00
Lambert 2001	[181]	*P. boldus, T. foenum-gracum*	warfarin	minor	increased INR	0.80
Lantz 1 1999	[182]	*Hypericum perforatum*	sertraline	minor	serotonin syndrome	0.80
Lantz 2 1999	[182]	*Hypericum perforatum*	sertraline	minor	serotonin syndrome	0.70
Lantz 3 1999	[182]	*Hypericum perforatum*	sertraline	severe	serotonin syndrome	0.70
Lantz 4 1999	[182]	*Hypericum perforatum*	sertraline	severe	serotonin syndrome	0.80
Lantz 5 1999	[182]	*Hypericum perforatum*	nefazodone	minor	serotonin syndrome	0.60
Lee 2004	[12]	*Aloe barbadensis*	sevoflurane	severe	bleeding	0.80
Lee 2006	[60]	royal jelly	warfarin	severe	bleeding	0.91
Leung 2008	[183]	*Lycium barbarum*	warfarin	minor	increased INR	0.80
Mai 2000	[184]	*Hypericum perforatum*	cyclosporine	minor	decreased level	0.50

Document	Ref.	Latin name	Drug	Severity	Reaction	RI*
Mateo 2012	[185]	*Panax ginseng*	raltegravir	severe	increased level	0.90
McGovern 2010	[186]	*Rhodiola rosea*	escitalopram	severe	palpitations	0.60
McRae 1996	[187]	*Eleuthrococcus senticosus*	digoxin	minor	increased level	0.91
Meisel 2008	[188]	*Ginkgo biloba*	ibuprofen	fatal	bleeding, intracerebral	0.70
Melchardt 2014	[59]	multiple herbs	temozolamide	severe	hepatotoxicity	0.90
Mendoza 2004	[189]	unspecified mixture	warfarin	severe	low INR AMI	0.50
Mergenhagen 2008	[13]	*Vaccinium spp*	warfarin	minor	Increased INR	0.90
Myers 2015	[190]	Ginseng NOS	lamotrigine	severe	DRESS	0.60
Naccarato 2012	[191]	*Ginkgo biloba* multiherb	efavirenz	minor	increased viral load	0.80
Nayeri 2017	[192]	*Curcuma longa*	tacrolimus	severe	increased level	0.90
Nebel 1999	[193]	*Hypericum perforatum*	theophylline	minor	decreased level	0.90
Nowak 2005 1[a]	[194]	*Matricaria recutita,* multiherb	cyclosporine	minor	decreased cyclosporine	0.82
Nowak 2005 2[a]	[194]	Chamomile NOS	cyclosporine	minor	increased cyclosprine	0.30
Nowak 3 2005	[194]	*Hibiscus spp* multiherb	cyclosporine	minor	decreased cyclosporine	0.70
Orr 2013	[195]	*Trifolium pratense*	methotrexate	mild	GI symptoms	0.50
Paeng 2007	[196]	*Vaccinium spp*	warfarin	minor	increased INR	0.80
Page 1999	[197]	*Angelica sinensis*	warfarin	minor	increased INR	0.90
Porretaz 2017	[198]	*Hibiscus spp*	erlotinib	severe	severe cutanous ADE	0.70
Prasad 2008	[199[	multiherbal	sertraline	minor	therapeutic failure	0.91
Rindone 2005	[200]	*Vaccinium spp*	warfarin	severe	bleeding	0.70
Rivera 2012	[201]	*Lycium barbarum*	warfarin	severe	bleeding	0.80
Rosado 2003	[202]	Ginseng NOS	warfarin	severe	thrombosis	0.50
Rozenfeld 2004	[203]	Glucosamine chondroitin	warfarin	minor	Increased INR	0.90
Rubin 2006	[204]	*P methysticum, V. officinalis*	paroxetine	severe	serotonin syndrome	0.64
Ruschitska 2000	[205]	*Hypericum perforatum*	cyclosporine	severe	decreased level, rejection	0.70
Schwarz 2003	[206]	*Hypericum perforatum*	oral contraceptive	severe	reduced efficacy	0.70
Segal 2006	[207]	*Matricaria chamomilla*	warfarin	severe	additive	0.50
Sobieraj 2010	[19]	*Opuntia spp*	glipizide, metformin	mild	hypoglycemia	0.90
Spinella 2002	[61]	*G.biloba , H. perforatum*	buspirone, fluoxetine	mild	serotonin syndrome	0.80
Strippoli 2013	[208]	*Aronia melocarpum*	trabectedin	severe	rhabdomyolysis	0.90
Su 2010	[7]	*P.ginseng, Liriope, Schisandra*	warfarin	severe	Bleeding intracerebral	0.70
Suvarna 2003	[209]	*Vaccinium spp*	warfarin	fatal	GI and pericardial bleeding	0.40

Document	Ref.	Latin name	Drug	Severity	Reaction	RI*
Tam 1985	[210]	*Salvia miltiorrhiza*	warfarin, salicylate	severe	bleeding	0.70
Tamura 2012	[211]	Melilot, NOS	interferon 1b	severe	hepatotoxicity	0.50
Taylor 1999	[212]	*Camellia sinensis*	warfarin	minor	increased INR	0.70
Vandenbout 2008	[213]	multiherbal	lopinavir	mild	diarrhea, increased levels	0.09
Vanstrater 2012	[214]	*Hypericum perforatum*	clozapine	mild	emergence of schizophrenia	0.80
Walsh 2005	[215]	*Vaccinium spp*	warfarin	minor	increased INR	0.40
Welch 2007	[216]	*Vaccinium spp*	warfarin	minor	increased INR	0.80
Werba 2008	[217]	*Camellia sinensis*	simvastatin	minor	muscle pain	1.00
Wiegman 2009	[218]	*Ginkgo biloba*	efavirenz, emtracitabine, tenofovir	severe	treatment failure	0.70
Wong 2003	[219]	multiherbal	warfarin	severe	bleeding	0.91
Yu 1997	[218]	*Salvia miltiorrhiza* multiherb	warfarin	severe	bleeding	0.60

Table S5. Methods and herbal materials investigated in clinical studies.

Reference	Methods	Herbal experimental materials
Abdul 2008 [77]	An open-label, three-treatment, randomized crossover clinical trial was undertaken and involved 12 healthy male subjects of known CYP2C9 and VKORC1 genotype. A single dose of warfarin was administered alone or after 2 weeks of pretreatment with either garlic or cranberry. Warfarin enantiomer concentrations, INR, platelet aggregation and clotting factor activity were measured to assess pharmacokinetic and pharmacodynamic interactions between warfarin and herbal medicines.	Botanical study materials included GNC cranberry juice concentrate labelled to contain 500 mg of *Vaccinium spp.* (cranberry) juice concentrate dose equivalent to 57g of dried fruit/day and *A. sativum* as Garliplex™ 2000 enteric-coated garlic tablets, labelled as containing 2000 mg of fresh garlic bulb equivalent to 3.71 mg of allicin per tablet dosed twice daily.

Al-Jenoobi 2015 [20]	Effect of *Curcuma longa* rhizome powder and its ethanolic extract on CYP2D6 and CYP3A4 metabolic activity was investigated in vitro using human liver. In phase-I, six healthy human subjects received a single dose (30 mg) of DEX syrup, and in phase-II DEX syrup was administered with Curcuma powder. The enzyme CYP2D6 and CYP3A4 mediated O- and N-demethylation of dextromethorphan into dextrorphan (DOR) and 3-meth- oxymorphinan (3-MM), respectively.	Locally purchased rhizome. 1.5g dried, powdered, and extracted with ethanol.
Andrén 2007 [102]	Sixteen patients with hypercholesterolemia treated with a stable dose of atorvastatin (10–40 mg/daily) for at least 3 months were treated with Movina™ one tablet (containing 300 mg of *Hypericum perforatum*) twice daily and control (a commercially available multivitamin tablet (Vitamineral™). After a run-in period of 4 weeks, patients were randomised to treatment with either Movina™ or control for 4 weeks in a crossover design. The atorvastatin dose was kept unchanged during the study period (12 weeks), and assessments of total cholesterol, HDL cholesterol, LDL cholesterol and triglyceries were performed in the morning with the patients in the fasting condition. The difference between control and active treatment in LDL cholesterol after 4 weeks of treatment was the primary endpoint.	Commercial tablet (Movina™) labelled as 300mg *Hypericum perforatum*.
Aruna 2007 [91]	A randomized, open-label, crossover study of 10 healthy male volunteers was performed to evaluate different doses of *Ginkgo biloba* on cilastazol and clopidogrel pharmacodynamics. The dosage schedules were 120 mg *G. biloba*, 240 mg *G. biloba*, 100 mg cilostazol, 200 mg cilostazol, 75 mg clopidogrel, 150 mg clopidogrel, 120 mg *G. biloba* + 100 mg cilostazol and 120 mg *G. biloba* + 75 mg clopidogrel. Platelet aggregation, platelet count, bleeding time and clotting time were measured 0 and 6 h after drug administration. Platelet aggregation was performed using a dual channel aggregometer.	Not specified
Bedada 2016 [130]	The open-label, two period, sequential study was conducted in 12 healthy human volunteers to evaluate the impact of resveratrol (RSV) 500mg pretreatment on CYP2E1 enzyme activity and pharmacokinetics of chlorzoxazone (CHZ) in healthy human volunteers. A single dose of RSV 500 mg was administered once daily for 10 days during treatment phase. A single dose of CHZ 250 mg was administered during control and after treatment phases under fasting conditions. The blood samples were collected after CHZ dosing at predetermined time intervals and analyzed by HPLC (high performance liquid chromatography). .	Zenith Nutritions (Bangalore, India)

bell 2007 [103]	This open-label fixed-schedule investigation performed in 10 healthy men consisted of 2 days of prednisone single-dose administration and blood sampling, which were separated by 4 weeks of *Hypericum perforatum* 300mg thrice daily. Prednisone and predisolone pharmacokinetic parameters served as end points.	*Hypericum perforatum* standardized to hypericin 03%) tablets from HBC Protocols (SantaMonica,CA)
bell 2007 [104]	Eight male subjects participated in this single-dose study to determine the pharmacokinetics of ibuprofen before and after 21 days of *Hypericum perforaturm* 300 mg thrice daily. Plasma ibuprofen concentrations were determine using a stereoselective reversed phase HPLC assay.	Source not specified
Chen 2012 [120]	This trial examined the Traditional Chinese Medicine fomulation known as LIu Wei Di Huang Wan (LDW) on the activities of CYP2C19, CYP2D6 and CYP3A4 in 12 Chinese healthy subjects in a single center, controlled, non-blinded, two-way crossover clinical design. The subject pool consisted of six extensive metabolizers with CYP2C19*1/*1 and six poor metabolizers with CYP2C19*2/*2. Placebo or 4.8g LDW (12 pills, 0.2g/pill, twice daily) was given to each participant for 14 continuous days with a wash-out period of 2 weeks after an oral administration of 30mg omeprazole, 30mg dextromethorphan hydrobromide and 7.5mg midazolam. The activities of CYP2C19, CYP2D6 and CYP3A4 were ascertained by their respective plasma or urinary metabolic ratios on day 14 post-treatment. Liu Wei Di Huang Wan (LDW) is a Traditional Chinese formulation that contains *Rehemannia, Alismu, Cornus, Paeonia* (moutan), *Dioscorea,* and *Poria* (hoelen).	The Traditional Chinese Medicine formulation known as Liu Wei Di Huang Wan produced by Jiuzhitang Co., Ltd (Changsha, Hunan, China) containing 160 g *Rehmannia,* 60 g *Alisma,* 80 g *Ccornus*, 60 g moutan, 80 g *Dioscorea* and 60 g hoelen was crushed into a fine powder and mixed well, and 35 to 50 g of refined honey and water were added per 100 g powder, then dried and divided into 0.2 g per pill. The quality control required that the paeonol content be about 2.1 mg/g (±4%,).
Dai 2013 [92]	This open-label, randomized, two-period, two-treatment, balanced, crossover study was performed in 14 healthy men. Subjects received simvastatin 40 mg once daily, co-treated with placebo or *Ginkgo biloba* extract 120 mg twice daily. Each treatment was administered for 14 days, separated by a wash-out period of 1 month. Simvastatin, simvastatin acid and lipoprotein concentrations were assessed.	*Ginkgo biolba extract* (GBE) 120mg tablet, Yangzijiang Pharmaceutical, China

Fan 2007 [121]	Eighteen healthy men, including 6 CYP2C19*1/*1, 6 CYP2C19*1/*2 or *3 and 6 CYP2C19*2/ *2 were enrolled in a 2-phase, randomized, crossover clinical trial. In each phase, the volunteers received either placebo or 10 mL of the Traditional Chinese Medical Formulation Yin Zhi Huang (YZH) oral liquid, 3 times daily for 14 d. Then all the patients took a 20 mg omeprazole capsule orally. Blood samples were collected up to 12 h after omeprazole administration. Plasma concentration of omeprazole and its metabolites were quantified by HPLC with UV detection. Yin Zhi Huang contains *Artemisia scoparia, Scutellaria baicalensis,* and *Lonicerae japonica*.	YZH oral liquid was purchased from Shuang He (Beijing, China)
Fan 2008 [81]	Eighteen unrelated healthy men who were CYP2C9*1/*1 with different OATP1B1 haplotypes (six OATP1B1*1b/*1b, six OATP1B1*1b/*15, and six OATP1B1*15/*15) were selected to participate in this study. Rosuvastatin (20 mg orally) pharmacokinetics after coadministration of placebo and 50-mg baicalin tablets (three times daily orally for 14 days) were measured for up to 72 h by liquid chromatography–mass spectrometry in a two-phase randomized crossover study.	Tableted extract by Sheng Tai Chun Pharmaceutical, Xiangtan, Hunan, China
Fan 2009 [93]	Ten unrelated healthy male volunteers participated in a 3-stage sequential study to determine the effects of *Ginkgo biloba* extract (GBE) 360mg/day on plasma concentrations of talinolol measured by high-performance liquid chromatography after talinolol 100 mg was administrated alone, with a single oral dose of GBE (120 mg), and after 14 days of repeated GBE ingestion (360 mg/day).	*Ginkgo biloba* 120 mg standardized extract; Rui Bang Pharmaceutical Co., Jiangsu, China
Fan 2011 [105]	In a two-phase, randomized, crossover study with a 4-week washout period between phases, 15 healthy men with specific solute carrier organic anion transporter family member 1B1 (SLCO1B1) genotypes were given pretreatment with *Hypericum perforatum* 325 mg or placebo three times daily for 14 days, and a single dose of repaglinide 1 mg was administered followed by 75 g glucose at 15 minutes after repaglinide administration to determine impact of Hypericum perforatum on CYP2C8 activity.	Nutraceutical Corp. for Solaray, Inc., Park City, UT, USA

Fatima 2014 [129]	This was a randomized open label crossover study of 10 type II diabetic patients. The dosage schedules were either single dose of 500 mg *Phyllanthus emblica* extract or 75 mg clopidogrel or 75 mg aspirin or 500 mg *P. emblica* + 75 mg clopidogrel or 500 mg *P. emblica* + 75 mg aspirin. After single dose study and washout period, patients received either 500 mg P. emblica extract twice daily or 75 mg clopidogrel or 75 mg aspirin once daily or combinations for 10 days. Platelet aggregation was measured at baseline and at 4 h of treatment after single and multiple dose study along with recording of bleeding and clotting time.	CAPROS® capsules were supplied by Natreon, Inc., New Brunswick, NJ, USA. CAPROS® is an aqueous extract of the edible fruits of *Phyllanthus emblica* (Amla), containing not less than 60% of low molecular weight hydrolysable tannins comprising Emblicanin-A, Emblicanin-B, Punigluconin and Pedunculagin as the bioactives.
Fuhr 2007 [131]	Sixteen healthy males were enrolled in this single-center open-label cross-over design with period-balanced random allocation of sequence. Immediate release nifedipine (10mg) was administered as a 3A4 marker alone or in combination with 280mg silymarin 280mg 10 hours and 1.5 hours prior to a nifedipine dose.	Legalon, Madaus GmbH; Cologne, Germany. Each capsule contains 173-186.9mg dry extract from *Silybum marianum* (milk thistle) fruits equivalent to 140mg silymarin
Goey 2014 [107]	The pharmacokinetics of docetaxel (135 mg administered intravenously over 60 min) were compared in 10 cancer patients before and after 14 days of supplementation with *Hypericum perforatum* (300 mg extract [Hyperiplant] three times daily) in an open-label non-randomized crossover trial.	*Hypericum perforatum* 300mg extract standardized to 0.36–0.84 mg hypericin and 9–19 mg hyperforin; VSM Geneesmiddelen BV, Alkmaar, The Netherlands
Guo 2012 [94]	In an open-label design, sixteen volunteers received a single oral dose of 40 mg atorvastatin, followed by a wash-out period of at least 5 days. Then the volunteers took 360 mg *Ginkgo biloba* extract (GBE) daily for 14 days, followed by a single dose of 40 mg atorvastatin. Serial blood samples obtained over a period of 48 h after atorvastatin ingestion were subjected to determination of atorvastatin plasma concentrations and markers of cholesterol synthesis (lathosterol) and cholesterol absorption (sitosterol)	Dr Willmar Schwabe GmbH & Co. Germany

Gurley 2002 [78]	Twelve healthy volunteers (6 females) were randomly assigned to receive either *Hypericum perforatum* 300mg thrice daily, *Allium sativum* oil 500mg thrice daily, *Panax ginseng*, 500mg thrice daily or *Ginkgo biloba* 60mg four times daily for 28 days in an open-label design randomized for cross-over sequencing. For each subject, a 30-day washout period was interposed between each supplementation phase. Probe-drug cocktails of midazolam, caffeine, chlorzoxazone, and debrisoquin (INN,debrisoquine) were administered before supplementation (baseline) and at the end of supplementation.	Supplements purchased from Wild Oats Marets Inc, Boulder CO icluded: *Allium sativum* oil 500mg, *Ginkgo biloba* standardized to 24% flavone glycosides and 6% terpene lactones, *Hypericum perforatum* standardezed to 0.3% hypericine and *Panax ginseng* standardized to 5% ginsenosides.
Gurley 2005 [75]	Twelve healthy volunteers (6 females) were assigned in random sequence in this open label trial to receive *Hydrastis canadensis* (goldenseal) 900mg thrice daily, *Actea racemosa* (black cohosh) 1090mg twice daily, kava kava 1000mg twice daily, or valerian 125mg thrice daily for 28 days. For each subject, a 30-day washout period was interposed between each supplementation phase. Probe drug cocktails of midazolam and caffeine, followed 24 hours later by chlorzoxazone and debrisoquine were administered before (baseline) and at the end of supplementation. Pre- and post-supplementation phenotypic trait measurements were determined for CYP3A4/5, CYP1A2, CYP2E1, and CYP2D6 using 1-hydroxymidazolam/ midazolam serum ratios (1-hour sample), paraxanthine/caffeine serum ratios (6-hour sample), 6-hydroxychlorzoxazone/chlorzoxazone serum ratios (2-hour sample), and debrisoquine urinary recovery ratios (8-hour collection), respectively	*H. canadensis* (lot # 303415) and *P. methysticum* (lot #V4694K06) with no standardization claims were purchased from the same vendor (Wild Oats Markets, Inc. Boulder, CO.). The *A. racemosa* supplement (lot #060706) was a product of Solaray Inc. (Park City, UT) standardized to 0.2% triterpene glycosides, and the *V. officinale* supplement (lot #303990) was manufactured by Vitamer (Lake Forest, CA) without standardization claim.
Gurley 2005 [79]	Twelve healthy volunteers with extensive CYP2D6 phenotype between the ages of 60 and 76 (mean = 67 years) were assigned in random sequence to receive *A. sativum* oil 500mg thrice daily, *P. ginseng* 500mg thrice daily, *G. biloba* 60mg four times daily, or *H. perforatum* 300mg thrice daily in an open-label fashion for 28 days followed by a 30-day washout period. Probe drug cocktails of midazolam, caffeine, chlorzoxazone, and debrisoquine were administered before and at the end of supplementation. Pre- and post-supplementation phenotypic ratios were determined for CYP3A4, CYP1A2, CYP2E1, and CYP2D6 using 1-hydroxymidazolam/midazolam serum ratios (1-hr), paraxanthine/caffeine serum ratios (6-hr), 6-hydroxychlorzoxazone/ chlorzoxazone serum ratios (2-hr), and debrisoquine urinary recovery ratios (8-hr), respectively. The content of purported "active" phytochemicals was determined for each supplement.	Botanical experimental materials were purchased from Vitamer, Lake Forest CA including *A. sativum* oil 500mg capsules, *G. biloba* standardized to 24% flavone glycosides and 6% terpene lactones, *H perforatum standardized to 0.3% hypericin,* and *P. ginseng* standardized to 5% ginsenosides.

Gurley 2007 [100]	Twenty healthy volunteers were assigned in random sequence to receive a standardized *H. canadensis* (3210 mg daily) or *P. methysticum* (1227 mg daily) supplement in an open-label fashion for 14 days, followed by a 30-day washout period. Subjects were also randomized to receive rifampin (600 mg daily, 7 days) and clarithromycin (1000 mg daily, 7 days) as positive controls for P-gp induction and inhibition, respectively. Digoxin (Lanoxin®, 0.5 mg) was administered orally before and at the end of each supplementation and control period. Serial digoxin plasma concentrations were obtained over 24 hours and analyzed by chemiluminescent immunoassay. Comparisons of AUC (0–3), AUC (0–24), Cmax,, CL/F, and elimination half-life were used to assess the effects of goldenseal, kava kava, rifampin, and clarithromycin on digoxin pharmacokinetics.	Botanical experimental materials included *Hydrastis canadensis* purchased from Nature's Resource Products, Mission Hills, CA. standardized to contain 24.1 mg isoquinoline alkaloids and *Piper methysticum* purchased from Gaia Herbs, Brevard, NC. standardized to contain 75 mg kavalactones.
Gurley 2006 [76]	Serial plasma concentration-time profiles of the P-gp substrate, digoxin, were used to determine whether supplementation with *Silybum marianum* (milk thistle) or *Actea racemosa (*black cohosh) modified P-gp activity in vivo. Sixteen healthy volunteers were assigned in random sequence to receive a standardized milk thistle (900 mg daily) or black cohosh (40 mg daily) supplement in an open-label fashion for 14 days, followed by a 30-day washout period. Subjects were also randomized to receive rifampin (600 mg daily, 7 days) and clarithromycin (1000 mg daily, 7 days) as positive controls for P-gp induction and inhibition, respectively. Digoxin (Lanoxicaps®, 0.4 mg) was administered orally before and at the end of each supplementation and control period.	Botanical experimental materials were purchased from Enzymatic Therapy, Inc. Green Bay, WI. Including *A. racemosa* standardized to 2.5% triterpene glycosides and *S. marianum* standardized to 80% silymarin
Gurley 2008 [101]	Sixteen healthy volunteers were assigned in random sequence to receive either *Hydrastis canadensis* root extract 1323mg thrice daily or *Piper methysticum* rhizome extract 1227mg thrice daily in an open-label fashion for 14 days. Each supplementation phase was followed by a 30-day washout period. Midazolam (8 mg, per os) was administered before and after each phase, and pharmacokinetic parameters were determined using standard non-compartmental methods	*Hydrastis canadensis* was purchased from Nature's Resource Products, Mission Hills, CA. standardized to contain 24.1 mg isoquinoline alkaloids and *Piper methysticum* standardized to 75mg kavalactones from Gaia Herbs, Brevard, NC.

Heger 2014 [119]	A double-blind, randomised, 2-period crossover study was performed to investigate the effects of L*avendula angustivolia* oil (Silexan) on the pharmacokinetics and pharmacodynamics of a combination oral contraceptive containing ethinyl estradiol 0.03 mg (EE) and levonorgestrel 0.15 mg (LNG) in healthy, fertile, adult females. During 2 consecutive cycles of 28 days, oral contraception was given for 21 days combined with 1 9 160 mg/day Silexan or placebo. Plasma concentration–time profiles of EE and LNG were obtained on day 18 ± 1 up to 24 h after dosing. The primary outcome measure was the area under the concentration–time curve over a dosing interval of s = 24 h (AUCs)	160mg Lavender oil encapsulated as silexan, the active substance of Lasea produced by Dr. WIllmar Schwabe GmbH & Co. Karlsruhe, Germany
Hennessy 2002 [108]	Twenty-two healthy volunteers were randomized in this single-blind study to receive either a standardized extract of *Hypericum perforatum* containing 600mg three times daily (n=15) or placebo (N=7) for 16 days. P-glycoprotein expression and rhodamine efflux were used to measure p-glycoprotein activity.	Not specified
Hu 2013 [82]	Twelve healthy male volunteers were sequentially randomized in this open-label cross-over trial to take a single dose pitavastatin 2 mg orally with water or with 200ml double strength *Citrus paradisi* (grapefruit) juice on separate occasions and plasma concentration of pitavastatin acid and lactone were measured over 48 h.	Not specified
Ikehata 2008 [86]	An open-label and randomized crossover study was performed at 2-week intervals. In the control experiment, after a 10 h overnight fast, 10 mg of nifedipine (Adalat® capsule) was administered orally and blood was collected at 0, 0.5, 1, 2, 3, 4, 5, 6, and 8 h. In the combination experiment, the volunteers were orally administered 10 mg of nifedipine together with six tablets containing concentrated turmeric extract (480 mg of curcuminoid per six tablets), which is the general daily dose followed by blood sampling.	Junsei-Nosyuku-Ukon-Tsubu tablets by Yamada Health Partners, Inc. (Tokyo,Japan). Each tablet contains curcuminoids (80 mg), reduced maltose, crystallized cellulose, dietary fiber, black pepper extract, selenium yeast, colzaoil, and shellac.
Jiang 2004 [109]	An open-label, three-way crossover randomized study performed in 12 healthy male subjects, who received a single 25-mg dose of warfarin alone or after 14 days' pretreatment with *Hypericum perforatum* (St. John's wort), or 7 days' pretreatment with Panax ginseng. Dosing with St John's wort or ginseng was continued for 7 days after administration of the warfarin dose. Platelet aggregation, international normalized ratio (INR) of prothrombin time,warfarin enantiomer protein binding, warfarin enantiomer concentrations in plasma and S-7-hydroxywarfarin concentration in urine were measured.	Study botanicals included Bioglan standardized dry extract equivalent to 1 g *Hypericum perforatum* flowering herb top, 0.825 mg hypericin and 12.5 mg hyperforin and Golden Glow Korean ginseng tablets each equivalent to 0.5g Panax ginseng root and 8.93mg gensenosides as gensenoside Rg1.

Jiang 2006 [95]	S-warfarin concentration and response (prothrombin complex activity) data from healthy male subjects (n = 24) who received a single warfarin dose (25 mg) and *Hypericum perforatum* (St John's wort). *Panax ginseng* (Asian ginseng), *Ginkgo biloba*, or ZIngiber *officinale* (ginger) in a randomized open-label fashion with at least a 14-day wash out between treatments. Herbal treatment duration was not specified. Data analysis included use of a population pharmacokinetic-pharmacodynamic modeling approach.	Product and dose not specified
Jiang 2010 [53]	Patients were administered tacroimus during phase 1 and then *Schisandra sphenanthera* 22.5mg thrice daily was provided in phase 2. During the first phase of treatment, 46 patients received the same oral dose of tacrolimus. In the second phase of treatment, 21 patients (Group A) received the same dose of tacrolimus, and 25 patients (Group B) received a lower dose of tacrolimus. The concentration of Tac in the blood and the biochemical indices of liver function, as well as symptoms of Tac-related toxicity, were determined, and 14 patients were selected for a pharmacokinetic study in a non-randomized fashion.	Each capsule contained 11.25 mg of deoxyschizandrin; Hezheng Pharmaceutical Company, Chengdu, China
Suzuki 2014 [132]	Nine healthy patients underwent analysis of CYP1A2, CYP2C9, CYP2D6, and CYPA4/5 activity via sampling after a drug probe cocktail before and after 14 days of a standardized *Silybum marianum* supplement containing 175 mg dried extract of milk thistle achenes three times daily. This study was open-label and fixed sequence.	Legalon 140 capsules; MADAUS GmbH, Cologne, Germany. Each capsule contains 175 mg dried extract of milk thistle achenes, or 140 mg silymarin representing silybin A (21.2 mg), silybin B (29.5 mg), isosilybin A (11.4 mg), isosilybin B (8.2 mg), silychristin (31.5 mg), silydianin (36.4 mg), and taxifolin (5.9 mg).

Kim 2010 [122]	A two-way crossover clinical trial with a 2-week washout period was conducted in 14 healthy volunteers. In phases I and II, subjects received 150 mg bupropion with or without woohwangcheongsimwon suspension four times (at -0.17, 3.5, 23.5 and 47.5 h, with the time of bupropion administration taken as 0 h) in a randomized balanced crossover order. Bupropion and 4-hydroxybupropion plasma concentrations were measured for up to 72 h by LC-MS/MS. Urine was collected up to 24 h to calculate the renal clearance. Woohwangcheongsimwon is a multibotanical product containing Bovine hoof, *Dioscoreae rhizome*, Glycyrrhizae root and rhizome, ginseng (species not specified) root, Typha Pollen, Massa medicata fermentata (flour, wheat bran, rice bean powder, and bitter apricot seed powde fermented in aqueous extract of A*rtemisia centifolia, Polygonum hydropiper* L., and *Xanthium sibiricum*), *Glycine* seed, *Cinnamomum* cortex, *Paeoniae* root, *Liriopsis* tuber, *Scutellaria* root, *Angelica* root, *Saposhnicoviae* root, *Atractylodis* rhizome, *Bupleurum* root, *Platycodonis* root, *Aermica* seed, *Poria* mycelium, *Cnidii* rhizome, Civet musk, *Antelopis* Cornu, Borneolum (borneol and isoborneol), *Ampelopsi*s root, and *Zingiberis* rhizome.	Kwang-Dong Pharmaceutical Company, Seoul, Korea
Kim 2010 [96]	An open-label, randomized, 2-period, 2-treatment, 2-sequence, single-dose crossover study was conducted in 24 healthy Korean male volunteers. All volunteers were randomly assigned to a sequence group for the 2 treatments, which consisted of ticlopidine 250 mg alone and ticlopidine 250 mg with *Ginkgo biloba* extract 80 mg, separated by a 1-week washout period between the treatments. Bleeding time was determined just before dosing and at 5, 12, and 48 hours after dosing. Platelet aggregation was evaluated before dosing and at 4, 8, 26, and 48 hours after dosing. Blood samples (8 mL) from each of the volunteers were collected from an in-dwelling intravenous cannula inserted into a forearm vein before dosing and after dosing. Ticlopidine concentrations were determined by a validated method using HPLC and ultraviolet detection.	Yuyu Pharma, Inc., Jecheon-si Chungcheongbuk-do, Korea 24% glycosidic flavonoids and 6% terpinoids

Kim 2014 [220]	A randomized, double-blind, two-way crossover study was conducted with 34 healthy Korean subjects. All subjects were given an oral dose of cilostazol (100 mg) plus *Ginkgo biloba* extract (GBE 80 mg) or cilostazol (100 mg) plus placebo twice daily for 7 days. Plasma concentrations of cilostazol and its active metabolites (3,4-dehydrocilostazol and 4′-trans-hydroxycilostazol) were measured using liquid chromatography–tandem mass spectroscopy on day 7 for pharmacokinetic assessment. The adenosine diphosphate-induced platelet aggregation and bleeding time were measured at baseline and on day 7 for pharmacodynamic assessment.	Ginexin®; SK Chemical Co., Seoul, Korea
Kim 2017 [87]	An open-label, three-treatment, fixed-sequence study was conducted wherein 20 mg of rosuvastatin was given to 13 healthy subjects on day 1, followed by 20 mg of rosuvastatin plus 300 mg of epigallocatechin-3-gallate (EGCG), a major ingredient of *Camellia sinensis (*green tea) on day 4. After a 10-day pretreatment of EGCG up to Day 14, they received rosuvastatin (20 mg) plus EGCG (300 mg) once again (Day 15). Blood samples for the pharmacokinetic assessments were collected up to 8 hours after each dose of rosuvastatin.	EGCG Teavigo™, caffeine-free, 94% pure crystalline EGCG, Healthy Origins, Pittsburgh, PA, USA
Lee 2009 [127]	Thirty-one warfarinized patients with stable INR participated in this randomized, double-blind, crossover study. One group initially received warfarin with 1 g of Korean red ginseng (*Panax ginseng)* extract for 6 weeks and then after a 3-week washout period, received warfarin and placebo (Treatment A). Alternative group received treatment in the opposite order (Treatment B). Blood samples were collected to measure INR and plasma warfarin levels.	Aqueous decoction of 6yr red Korean ginseng root
Loughren 2020 [110]	Healthy volunteers received a fentanyl fixed-dose infusion and an individually tailored target-controlled infusion on separate days, before and after 30-day *Hypericum perforatum* (300mg thrice daily; n = 8) or placebo control (n = 8) in a randomized parallel-group design. Fentanyl plasma concentrations, pupil diameter, analgesic response to experimental pain (cold pressor) subjective side effects, and cognitive effects were measured. Plasma fentanylconcentrations and changes in pupil diameter were subjected to pharmacokinetic–pharmacodynamic modeling.	300 mg Kira™ tablet; Lichtwer Pharma, Berlin, Germany
Malati 2012 [128]	Twelve healthy subjects (8 males) completed this open label, single sequence pharmacokinetic study. Healthy volunteers received single oral doses of midazolam 8 mg and fexofenadine 120 mg, before and after 28 days of *Panax ginseng* 500 mg twice daily. Midazolam and fexofenadine pharmacokinetic parameter values were calculated and compared pre-and post *P. ginseng* administration. Geometric mean ratios (post-ginseng/pre-ginseng).	Panax ginseng 500 mg capsules (Vitamer Laboratories, Irvine, CA

Markert 2015 [111]	Twenty healthy volunteers (10 CYP2C19 extensive, four poor and six ultrarapid metabolizers) received therapeutic doses of ambrisentan (5 mg qd po) for 20 days and concomitantly *Hypericum perforatum* 300mg po thrice daily for the last 10 days. To quantify changes of CYP3A4 activity, midazolam (3 mg po) as a probe drug was used.	Jarsin™ Cassella-med GmbH & Co. KG, Koln, Germany
Mohutsky 2006 [97]	Two open-label, crossover pharmacokinetic studies in twelve healthy subjects were performed using tolbutamide and diclofenac as probe CYP2C9 substrates to explore the impact of *Ginkgo biloba.*	Ginkgold™ standardized to 24% ginkgo flavone glycosides, 6% terpene lactones Nature's Way, Springville, UT
Molto 2012 [88]	Fifteen HIV-infected patients receiving antiretroviral therapy with etravirine (400 mg once daily) for at least 4 weeks were included in this open-label fixed-sequence study. *Echinacea purpurea* root/extract-containing capsules were added to the antiretroviral treatment (500 mg every 8 h) for 14 days. Etravirine concentrations in plasma were determined by high-performance liquid chromatography immediately before and periodically after a morning dose. Plasma levels of etravirine on day 0 and etravirine plus E. purpurea on day 14 were used to calculate etravirine pharmacokinetic parameters by noncompartmental analysis and compared between days 0 and 14 by means of the geometric mean ratio (GMR).	Aq extract air-dried mixed with lactose
Mueller 2009 [113]	Twenty healthy male volunteers received an SJW powder with low hyperforin content 500mg twice daily for 2 weeks in an open-lable single sequence cross-over. Midazolam plasma concentration time profiles were characterized after a single oral dose of 7.5 mg midazolam on the day before and on the 14th day of *Hypericum perforatum* medication. Low hyperforin content induction of 3A4 considered insignificant.	Encapsulated powder Kneipp Werke, Würzburg, Germany
Mueller 2005 [112]	Forty-two male, healthy volunteers were randomized into six parallel SJW medication groups with varying composition especially with regard to hyperforin content. Midazolam plasma concentration profiles were characterized after a single oral dose of 7.5 mg midazolam on the day before and on the 14th day of SJW medication. Low hyperforin content was associated with less induction.	Powdered methanolic extract with varying hyperforin content

Murphy 2005 [114]	Sixteen healthy women were treated with a low-dose OC (Loestrin 1/20) and a placebo for two consecutive 28-day cycles in a single-blind sequential trial. Treatment with *Hypericum perforatum* 300 mg three times daily was then added for two additional 28-day cycles. Outcomes compared between control and treatment cycles included the pharmacokinetics of norethindrone and ethinyl estradiol, daily bleeding diaries, follicle growth, changes in cervical mucus and progesterone levels drawn at 7- to 10-day intervals	Hypericum Buyers Club (HBC) standardized alcoholic extract
Nakao 2007 [123]	The effect of multiherbal Shoseiryt (TJ-19) 4.5g twice daily for 7 days on the activities of CYP1A2, CYP2D6, CYP3A, xanthine oxidase (XO), and N-acetyltransferase 2 (NAT2) in 37 healthy subjects was assessed. The subject pool consisted of 19 extensive metabolizers (EMs) with CYP2D6*Wild/ *Wild, and 18 intermediate metabolizers (IMs) with CYP2D6*10/ *10. Ratios of caffeine and dextromethorphan to metabolites were used to assess impact on enzymes. Shosieryuto contains *Pinellia ternata* tuber, *Glycyrrhiza uralensis* root, *Cinnamomum cassia* bark, *Schisandra chinensis* fruit, *Asiasarum siebold* root, *Paeonia lactiflora* root, *Ephedra sinica* herb, and processed *Zingiber officinale.*	TJ-19; Tsumura, Tokyo, Japan
Nicandro 2007 [83]	A single-treatment, one-period, three-phase, open-labeled study was performed to evaluate the ability of I'm-Yunity™ containing *Corolus versicolor* mycelium to inhibit or induce CYP3A4 in 12 healthy adult volunteers (8 women and 4 men) aged between 23 and 54 years through the use of a CYP3A4-specific assay, the erythromycin breath test (EBT).	Chinese Medicine Holdings LTD
Niemenen 2010 [105]	A placebo-controlled, randomized, cross-over with two phases at intervals of 4 weeks was conducted with 12 healthy participants to assess impact of *Hypericum perforatum* (Jarsin™) on oxycodone pharmacodynamics and pharmacokinetics. St John's wort 300mg or placebo was administered t.i.d. for 15 days and oral oxycodone hydrochloride 15 mg on day 14. Oxycodone pharmacokinetics and pharmacodynamics were compared after St John’s wort or placebo. Behavioural and analgesic effects were assessed with subjective visual analogue scales and cold pressor test. Plasma drug concentrations were measured from 0 to 48 h, behavioural and analgesic effects from 0 to 12 h.	Jarsin™ 300 mg, Klosterfrau, Berlin, Germany; dry extract of St John’s wort 3–6:1, extraction solvent methanol 80%, hyperforin range 2–6%

Park 2018 [124]	An open-label, fixed-sequence, two-period, two-treatment cross-over study was conducted in 20 healthy Korean subjects. In period I, the individuals received celecoxib capsule 200 mg once daily for 4 days. In period II, only Ojeok-san (14.47 g/pack, three times daily) was administered for 4 days, followed by co-administration with celecoxib for 4 daysThe blood samples for pharmacokinetic evaluation were collected for up to 48 hr after the administration of celecoxib in each study period. Ojeok-sak contains *Atractylodis* rhizome, *Ephedra*, *Citri reticulatae* peel, *Pinellia* rhizome, *Zingiber officinale*, *Cinnamomum* bark, *Angelica gigantis* root, *Jujuba f*ruit, *Glycyrrhiza root*, *Paeoniae* root, *Angelica dahurica* root, *Poria* (hoelen), *Aauranti* fruit, *Cnidii* rhizome, *Magnolia*, *Platycodi* root, and *Cyperi* rhizome.	Hanpoong Pharm & Foods Co., Ltd., Seoul, Republic of Korea
Piscitelli 2000 [115]	An open-label fixed-sequence study in eight healthy volunteers examined the impact of *Hypericum perforatum* 300mg thrice daily for 14 days on indinavir pharamcokinetics.	300 mg reagent grade tablets, lot 190217, Hypericum Buyers Club, Los Angeles, CA, USA
Portoles 2006 [116]	An open-label non-randomized fixed-sequence study in 12 healthy volunteers investigated the impact of *Hypericum perforatum* 300mt thrice daily for 14 days on the pharmacokinetics of a single dose of ivabradine 10mg.	
Salman 2010 [89]	In a placebo-controlled randomized single-blinded crossover study, the effect of a water-based extract of a 200 mg *Erycoma longifolia* dose on the pharmacokinetics of a single dose of propranolol (Inderal®) in 14 healthy non-smoker young males was examined. Propranolol plasma concentrations were determined using a validated high-performance liquid chromatography (HPLC) method.	An extract of 50mg air-dried non-specified plant part, non-specified origin of *E. longifola* mixed with lactose
Saruwatri 2012 [125]	In an open-label study, thirty-one healthy females were studied to evaluate the impact of the multiherbal product keishi-bukuryo-gan dosed at 3.75g twice daily for 7 days on cytochrome P450 (CYP) 1A2, CYP2D6, CYP3A, xanthine oxidase (XO) and N-acetyltransferase 2 (NAT2) using caffeine and dextromethrophan as markers. Keishi-bukuryo-gan is often used for menopausal symptoms and contains *Cinnamomum cassia* bark, *Paeonia lactiflora* root, *Prunus persica* kernel, *Poria cocos* mycelium, and *Paeonia suffruticosa* bark.	Tsumura & Co. (Tokyo, Japan)

Souriti 2016 [54]	A cohort of 30 vitamin B12-deficient patients with neurological symptoms received oral fixed dose of hydroxocobalamin 15 mg Hdrx daily for 10 days followed by 15 mg monthly in this observational study. Clinical benefits were evaluated on haematological and biochemical parameters, and neurological improvement at days 10 and 90 compared to day 0. In the discussion, the authors state the the Hdrx compound was combined with *Hibiscus sabdariffa* 250mg.	5 mg of Hydroxocobalamin and 250 mg of powdered aqueous extract of *Hibiscus sabdariffa*.
Tankanow 2003 [84]	A randomized, crossover trial with 8 healthy volunteers was performed evaluating digoxin 0.25 mg alone for 10 days and digoxin 0.25 mg with *Crataegus* special extract WS 1442 450 mg twice daily for 21 days.	*Crataegus* leaves with flowers; Dr. Willmar Schwabe Pharmaceuticals Karlsruhe, Germany
Thronicke 2017 [134]	A total of sixteen cancer patients undergoing treatment with checkpoint inhibitors (nivolumab (75%), ipilimumab (19%) or pembrolizumab (6%)), nine of whom received concomitant treatment with *Viscum album* in this pilot observational cohort study.	Product and dose not specified
Tocaciu 2018 [133]	This open label non-crossover study was performed in 20 female patients with active malignancy taking letrozole or tamoxifen (n = 10 for each group). Patients took oral fucoidan, given in the form of Maritech extract, for a 3-week period (500 mg twice daily). Trough plasma concentrations of letrozole, tamoxifen, 4-hydroxytamoxifen, and endoxifen were measured using HPLC-CAD (high-performance liquid chromatography charged aerosol detector), at baseline and after concomitant administration with fucoidan derived from Undaria pinnatifida.	Maritech extract Marinova Pty Ltd, Hobart, Australia derived from *Undaria pinnatifida*
Vanbreeman 2020 [99]	Sixteen peri- and postmenopausal women consumed the *Humlulus lupulus* (hops) extract twice daily for 2 weeks, and the pharmacokinetics of tolbutamide, caffeine, dextromethorphan, and alprazolam were evaluated before and after supplementation as probe substrates	Prenylated hop phenols prepared from hops at the University of Illinois at the Chicago/National Institutes of Health (UIC/NIH) dosed at 59.5mg twice daily
Walker 2006 [85]	In a randomised controlled trial, 79 diabetic patients were randomized to 1200mg/day *Crataegus* (hawthorne) extract (N=39) or placebo for 16 weeks. At baseline and outcome, a wellbeing questionnaire was completed, and blood pressure and fasting blood samples taken. A food frequency questionnaire estimated nutrient intake.	Product equivalent to 6 g of dried flowering tops Faros® 600 [LI 132, Lichtwer Pharma, Berlin] extract 3:1, standardised to 2.2% flavonoids

Wang 2014 [126]	This open-label, multi-dose, single-center, sequential, inpatient study evaluated the effects of a two-herb combination drug (T89, Danshen plus Sanqi) on the steady-state pharmacodynamics (PD) and pharmacokinetics (PK) of warfarin in 24 healthy volunteers. Twenty-three subjects attained a stable international normalized ratio (INR) by taking warfarin alone prior to 1-week of added-on use of T89. T89 consists of *Salvia miltiorrhiza* root, *Panax notoginseng* root, and borneol as an absorption enhancer.	T89, Tasly Pharmaceutical Co Ltd., Tianjin, China
Xiao 2012 [90]	Eighteen healthy adult male participants were enrolled in a two-phase randomized crossover design. In each phase, the participants received placebo or genistein 1000mg/day for 14 days. On the 15th day, midazolam and talinolol were administered and blood samples were obtained. Midazolam and talinolol pharmacokinetic parameter values were calculated and compared before and after genistein administration.	Western EHSY (Shanghai, China)
Xie 2005 [117]	In an open label fixed-sequence trial, thirty subjects received a single dose of 60 mg of fexofenadine, 5 mg of midazolam syrup, and 2 mg of midazolam intravenous infusion (6 hours after the oral dose of midazolam) on study days 1 and 11. All subjects took 300 mg of *Hypericum perforatum* 3 times a day for 10 days. Plasma and urine samples were taken for PK analyses of fexofenadine and midazolam.	Source not specified
Xu 2008 [118]	A sequential crossover two-treatment study was performed with at least a 4-day washout in 21 healthy subjects. Each received gliclazide (80 mg) either alone or during 15-day treatment with *Hypericum perforatum* extract 300mg thrice daily. The area under the plasma concentration–time curve (AUC), apparent clearance (CL/F) and elimination half-life (t1/2) of gliclazide and incremental changes in glucose and insulin AUC were compared.	Kira™, LI 160 extract, Lichtwer Pharma, Berlin, Germany
Zadoyan 2012 [98]	A single-center, open-label, randomized, three- fold crossover, cocktail phenotyping design was applied. Eighteen health subjects received in random order 8 days each of placebo twice daily, *Ginkgo biloba* extract EGb 761® 120 mg twice daily, and EGb 761® 240 mg in the morning and placebo in the evening. In the morning of day 8, administration was performed together with the orally administered cocktail containing caffeine, tolbutamide, omeprazole,midazolam, and dextromethorphan.	Ginkgo biloba extract EGb 761

Zhou 2013 [80]	A randomized, placebo-controlled, two-period crossover pharmacokinetic drug interaction study was conducted in 14 healthy Chinese men. Fexofenadine was used as a P-gp phenotyping probe to assess impact of *Astragalus* root extract granules 4 g twice daily or placebo twice daily for 7 days on fexofenadine plasma concentrations as determined by HPLC.	Decoction of root Sichuan Baili Pharm Co., Ltd (Sichuan, China)

References

1. Smith, T.; May, G.; Eckl, V.; Morton Reynolds, C. US Sales of Herbal Supplements Increase by 8.6% in 2019. *HerbalGram* **2020**, *127*, 54–69. Available online: http://cms.herbalgram.org/herbalgram/issue127/hg127-mktrpt-2019.html (accessed on 20 December 2020).
2. Martin, K.J.; Jordan, T.R.; Vassar, A.D.; White, D.B. Herbal and Nonherbal Alternative Medicine Use in Northwest Ohio. *Ann. Pharmacother.* **2002**, *36*, 1862–1869, doi:10.1345/aph.1a215.
3. Clarke, T.C.; Black, L.I.; Stussman, B.J.; Barnes, P.M.; Nahin, R.L. *Trends in the Use of Complementary Health Approaches among Adults: United States, 2002–2012 National Health Statistics Reports No. 79*; National Center for Health Statistics: Hyattsville, MD, USA, 2015.
4. Rashrash, M.; Schommer, J.C.; Brown, L.M. Prevalence and Predictors of Herbal Medicine Use Among Adults in the United States. *J. Patient Exp.* **2017**, *4*, 108–113, doi:10.1177/2374373517706612.
5. Low Dog, T. The use of botanicals during pregnancy and lactation. *Altern. Ther. Health Med.* **2009**, *15*, 54–58.
6. Percha, B.; Altman, R.B. Informatics confronts drug–drug interactions. *Trends Pharmacol. Sci.* **2013**, *34*, 178–184, doi:10.1016/j.tips.2013.01.006.
7. Su, Q.; Li, Y. Interaction between Warfarin and the Herbal Product Shengmai-Yin: A Case Report of Intracerebral Hematoma. *Yonsei Med. J.* **2010**, *51*, 793–796, doi:10.3349/ymj.2010.51.5.793.
8. Becker, B.; Greene, J.; Evanson, J.; Chidsey, G.; Stone, W. Ginseng-induced diuretic resistance. *JAMA* **1996**, *276*, 606–607.
9. Costa, M.L.; Rodrigues, J.A.; Azevedo, J.; Vasconcelos, V.; Eiras, E.; Campos, M.G. Hepatotoxicity induced by paclitaxel interaction with turmeric in association with a microcystin from a contaminated dietary supplement. *Toxicon* **2018**, *150*, 207–211, doi:10.1016/j.toxicon.2018.05.022.
10. Fasinu, P.S.; Bouic, P.J.; Rosenkranz, B. An Overview of the Evidence and Mechanisms of Herb–Drug Interactions. *Front. Pharmacol.* **2012**, *3*, 69, doi:10.3389/fphar.2012.00069.
11. Kupiec, T.; Raj, V. Fatal Seizures Due to Potential Herb-Drug Interactions with *Ginkgo biloba*. *J. Anal. Toxicol.* **2005**, *29*, 755–758, doi:10.1093/jat/29.7.755.
12. Lee, A; Chui, PT; Aun, CST; Gin, T; Lau, ASC. Possible interaction between sevoflurane and *Aloe vera*. *Ann. Pharmacother.* **2004,** 38, 1651–1654. doi: 10.1345/aph.1E098
13. Mergenhagen, K.A.; Sherman, O. Elevated International Normalized Ratio after concurrent ingestion of cranberry sauce and warfarin. *Am. J. Health Pharm.* **2008**, *65*, 2113–2116, doi:10.2146/ajhp080135.
14. Shaw, D.; Graeme, L.; Pierre, D.; Elizabeth, W.; Kelvin, C. Pharmacovigilance of herbal medicine. *J. Ethnopharmacol.* **2012**, *140*, 513–518, doi:10.1016/j.jep.2012.01.051.
15. Shetti, S.; Kumar, C.D.; Sriwastava, N.K.; Sharma, I.P. Pharmacovigilance of herbal medicines: Current state and future directions. *Pharmacogn. Mag.* **2011**, *7*, 69–73, doi:10.4103/0973-1296.75905.
16. Williamson, E.; Driver, S.; Baxter, K. (Eds.) *Stockley's Herbal Medicines Interactions*; Pharmaceutical Press: London, UK, 2009.
17. Russo, E.B. Taming THC: Potential cannabis synergy and phytocannabinoid-terpenoid entourage effects. *Br. J. Pharmacol.* **2011**, *163*, 1344–1364, doi:10.1111/j.1476-5381.2011.01238.x.
18. Weathers, P.J.; Arsenault, P.; Covello, P.S.; McMickle, A.; Teoh, K.H.; Reed, D. Artemisinin production in Artemisia annua: Studies in planta and results of a novel delivery method for treating malaria and other neglected diseases. *Phytochem. Rev.* **2010**, *10*, 173–183, doi:10.1007/s11101-010-9166-0.
19. Sobieraj, D.M.; Freyer, C.W. Probable Hypoglycemic Adverse Drug Reaction Associated with Prickly Pear Cactus, Glipizide, and Metformin in a Patient with Type 2 Diabetes Mellitus. *Ann. Pharmacother.* **2010**, *44*, 1334–1337, doi:10.1345/aph.1p148.

20. Al-Jenoobi, F.I.; Al-Thukair, A.A.; Alam, M.A.; Abbas, F.A.; Al-Mohizea, A.M.; Alkharfy, K.M.; Al-Suwayeh, S.A. Effect of *Curcuma longa* on CYP2D6- and CYP3A4-mediated metabolism of dextromethorphan in human liver microsomes and healthy human subjects. *Eur. J. Drug Metab. Pharmacokinet.* **2014**, *40*, 61–66, doi:10.1007/s13318-014-0180-2.
21. Awortwe, C.; Makiwane, M.; Reuter, H.; Muller, C.; Louw, J.; Rosenkranz, B. Critical evaluation of causality assessment of herb-drug interactions in patients. *Br. J. Clin. Pharmacol.* **2018**, *84*, 679–693, doi:10.1111/bcp.13490.
22. Chen, X.-W.; Sneed, K.B.; Pan, S.-Y.; Cao, C.; Kanwar, J.R.; Chew, H.; Zhou, S.- F. Herb-Drug Interactions and Mechanistic and Clinical Considerations. *Curr. Drug Metab.* **2012**, *13*, 640–651, doi:10.2174/1389200211209050640.
23. Aronson, J.K. Toward standardized reporting of drug interactions: The READI checklist for anecdotal reports. *Expert Rev. Clin. Pharmacol.* **2015**, *8*, 399–409, doi:10.1586/17512433.2015.1049598.
24. Ng, J.Y.; Munford, V.; Thakar, H. Web-based online resources about adverse interactions or side effects associated with complementary and alternative medicine: A systematic review, summarization and quality assessment. *BMC Med. Informatics Decis. Mak.* **2020**, *20*, 1–20, doi:10.1186/s12911-020-01298-5.
25. World Health Organization. The WHO Programme for International Drug Monitoring. In *Essential Medicines and Health Products*; WHO Website. 2015. Available online: who.int/medicines/areas/quality_safety/safety_efficacy/National_PV_Centres_Map/en/ (accessed on 7 January 2021).
26. United States Food and Drug Administration. FDA Adverse Event Reporting System (FAERS) Public Dashboard. 2021. Available online at: https://fis.fda.gov/sense/app/d10be6bb-494e-4cd2-82e4-0135608ddc13/sheet/7a47a261-d58b-4203-a8aa-6d3021737452/state/analysis (accessed on 28 October 2020).
27. Uppsala Monitoring Center. Vigibase. 2012. Available online: https://www.who-umc.org/vigibase/vigibase/ (accessed on 3 March 2021).
28. Awortwe, C.; Bruckmueller, H.; Cascorbi, I. Interaction of herbal products with prescribed medications: A systematic review and meta-analysis. *Pharmacol. Res.* **2019**, *141*, 397–408, doi:10.1016/j.phrs.2019.01.028.
29. Borse, S.P.; Singh, D.P.; Nivsarkar, M. Understanding the relevance of herb–drug interaction studies with special focus on interplays: A prerequisite for integrative medicine. *Porto Biomed. J.* **2019**, *4*, e15, doi:10.1016/j.pbj.0000000000000015.
30. Liu, D.; Zhang, L.; Duan, L.X.; Wu, J.J.; Hu, M.; Liu, Z.Q.; Wang, C.Y. Potential of herb-drug / herb interactions between substrates and inhibitors of UGTs derived from herbal medicines. *Pharmacol. Res.* **2019**, *150*, 104510, doi:10.1016/j.phrs.2019.104510.
31. Rombolà, L.; Scuteri, D.; Marilisa, S.; Watanabe, C.; Morrone, L.A.; Bagetta, G.; Corasaniti, M.T. Pharmacokinetic Interactions between Herbal Medicines and Drugs: Their Mechanisms and Clinical Relevance. *Life* **2020**, *10*, 106, doi:10.3390/life10070106.
32. Fugh-Berman, A.; Ernst, E. Herb-drug interactions: Review and assessment of report reliability. *Br. J. Clin. Pharmacol.* **2001**, *52*, 587–595, doi:10.1046/j.0306-5251.2001.01469.x.
33. VERBI Software. *MAXQDA 2020*; VERBI Software: Berlin, Germany, 2019.
34. Gnjidic, D.; Hilmer, S.; Blyth, F.M.; Naganathan, V.; Waite, L.; Seibel, M.; McLachlan, A.; Cumming, R.; Handelsman, D.J.; Le Couteur, D. Polypharmacy cutoff and outcomes: Five or more medicines were used to identify community-dwelling older men at risk of different adverse outcomes. *J. Clin. Epidemiology* **2012**, *65*, 989–995, doi:10.1016/j.jclinepi.2012.02.018.
35. Turner, J.P.; Jamsen, K.M.; Shakib, S.; Singhal, N.; Prowse, R.; Bell, J.S. Polypharmacy cut-points in older people with cancer: How many medications are too many? *Support. Care Cancer* **2016**, *24*, 1831–1840, doi:10.1007/s00520-015-2970-8.
36. *Lexicomp Online Database*; Lexicomp Inc.: Hudson, OH, USA, 2021. Available online: http://online.lexi.com. Subscription required to view (accessed on May 15, 2021).
37. Whirl-Carrillo, M.; McDonagh, E.M.; Hebert, J.M.; Gong, L.; Sangkuhl, K.; Thorn, C.F.; Altman, R.B.; Klein, T.E. Pharmacogenomics Knowledge for Personalized Medicine. *Clin. Pharmacol. Ther.* **2012**, *92*, 414–417, doi:10.1038/clpt.2012.96.
38. *IntegrativePro Drug-Nutrient Interaction Checker*. Integrative Therapeutics, LLC: Green Bay, WI, USA, 2021. Available online: https://www.integrativepro.com/drug-nutrient-interaction-checker (accessed on 15 May 2021).
39. Medscape Drug Interaction Checker. 2021. Available online: https://reference.medscape.com/drug-interactionchecker (accessed on 15 May 2021).

40. Memorial Sloan Kettering Cancer Center Search About Herbs. 2021. Available online: https://www.mskcc.org/cancer-care/diagnosis-treatment/symptom-management/integrative-medicine/herbs/search (accessed on 15 May 2021).
41. *Natural Medicines Online Database*. Therapeutic Research Center: San Francisco, CA, USA, 2021. Available online: https://naturalmedicines.therapeuticresearch.com (accessed on May 15, 2021).
42. IBM Corp. *IBM SPSS Statistics for Windows*; Version 27.0.; IBM Corp: Armonk, NY, USA, 2020.
43. Page, M.J.; McKenzie, J.E.; Bossuyt, P.M.; Boutron, I.; Hoffmann, T.C.; Mulrow, C.D.; Shamseer, L.; Tetzlaff, J.M.; Akl, E.A.; Brennan, S.E.; et al. The PRISMA 2020 statement: An updated guideline for reporting systematic reviews. *BMJ* **2021**, *372*, n71, doi:10.1136/bmj.n71. Available online: http://www.prisma-statement.org/ (accessed on 25 May 2021).
44. Ben Abacha, A.; Chowdhury, F.M.; Karanasiou, A.; Mrabet, Y.; Lavelli, A.; Zweigenbaum, P. Text mining for pharmacovigilance: Using machine learning for drug name recognition and drug–drug interaction extraction and classification. *J. Biomed. Informatics* **2015**, *58*, 122–132, doi:10.1016/j.jbi.2015.09.015.
45. Thompson, P.; Daikou, S.; Ueno, K.; Batista-Navarro, R.; Tsujii, J.; Ananiadou, S. Annotation and detection of drug effects in text for pharmacovigilance. *J. Cheminform.* **2018**, *10*, 1–33, doi:10.1186/s13321-018-0290-y.
46. Naranjo, C.A.; Busto, U.; Sellers, E.M.; Sandor, P.; Ruiz, I.; Roberts, E.A.; Janecek, E.; Domecq, C.; Greenblatt, D.J. A method for estimating the probability of adverse drug reactions. *Clin. Pharmacol. Ther.* **1981**, *30*, 239–245.
47. Horn, J.R.; Hansten, P.D.; Chan, L.-N. Proposal for a New Tool to Evaluate Drug Interaction Cases. *Ann. Pharmacother.* **2007**, *41*, 674–680, doi:10.1345/aph.1h423.
48. Council for International Organizations of Medical Sciences. Cumulative Pharmacovigilance Glossary. [Internet Site]. 2021. Available online: https://cioms.ch/wp-content/uploads/2021/03/CIOMS-Cumulative-Glossary_v1.1_3Jun2021.pdf (accessed on 15 June 2001).
49. Hill, A.; Van Der Lugt, J.; Sawyer, W.; Boffito, M. How much ritonavir is needed to boost protease inhibitors? Systematic review of 17 dose-ranging pharmacokinetic trials. *AIDS* **2009**, *23*, 2237–2245, doi:10.1097/qad.0b013e328332c3a5.
50. Alcamo, A.M.; Wolf, M.S.; Alessi, L.J.; Chong, H.J.; Green, M.; Williams, J.V.; Simon, D.W. Successful Use of Cidofovir in an Immunocompetent Child With Severe Adenoviral Sepsis. *Pediatrics* **2020**, *145*, e20191632, doi:10.1542/peds.2019-1632.
51. Khan, M.; Maryam, A.; Mehmood, T.; Zhang, Y.; Ma, T. Enhancing Activity of Anticancer Drugs in Multidrug Resistant Tumors by Modulating P-Glycoprotein through Dietary Nutraceuticals. *Asian Pac. J. Cancer Prev.* **2015**, *16*, 6831–6839, doi:10.7314/apjcp.2015.16.16.6831.
52. Meghwal, M.; Goswami, T.K. Piper nigrum and Piperine: An Update. *Phytother. Res.* **2013**, *27*, 1121–1130, doi:10.1002/ptr.4972.
53. Jiang, W.; Wang, X.; Xu, X.; Kong, L. Effect of *Schisandra sphenanthera* extract on the concentration of tacrolimus in the blood of liver transplant patients. *Int. J. Clin. Pharmacol. Ther.* **2010**, *48*, 224–229, doi:10.5414/cpp48224.
54. Souirti, Z.; Loukili, M.; Soudy, I.D.; Rtibi, K.; Özel, A.; Limas-Nzouzi, N.; El Ouezzani, S.; Eto, B. Hibiscus sabdariffaincreases hydroxocobalamin oral bioavailability and clinical efficacy in vitamin B12deficiency with neurological symptoms. *Fundam. Clin. Pharmacol.* **2016**, *30*, 568–576, doi:10.1111/fcp.12220.
55. Berretta, M.; della Pepa, C.; Tralongo, P.; Fulvi, A.; Martellotta, F.; Lleshi, A.; Nasti, G.; Fisichella, R.; Romano, C.; De Divitiis, C.; et al. Use of Complementary and Alternative Medicine (CAM) in cancer patients: An Italian multicenter survey. *Oncotarget* **2017**, *8*, 24401–24414, doi:10.18632/oncotarget.14224.
56. Knecht, K.; Kinder, D.; Stockert, A. Biologically-Based Complementary and Alternative Medicine (CAM) Use in Cancer Patients: The Good, the Bad, the Misunderstood. *Front. Nutr.* **2020**, *6*, 196, doi:10.3389/fnut.2019.00196.
57. Yeung, K.S.; Gubili, J.; Mao, J.J. Herb-Drug Interactions in Cancer Care. *Oncology* **2018**, *32*, 516–520.
58. Beauchamp, T.L.; Childress, J.F. *Principles of Biomedical Ethics*, 7th ed.; Oxford University Press: New York, NY, USA, 2019.
59. Melchardt, T.; Magnes, T.; Weiss, L.; Grundbichler, M.; Strasser, M.; Hufnagl, C.; Moik, M.; Greil, R.; Egle, A. Liver toxicity during temozolomide chemotherapy caused by Chinese herbs. *BMC Complement. Altern. Med.* **2014**, *14*, 115, doi:10.1186/1472-6882-14-115.
60. Spinella, M.; Eaton, L.A. Hypomania induced by herbal and pharmaceutical psychotropic medicines following mild traumatic brain injury. *Brain Inj.* **2002**, *16*, 359–367, doi:10.1080/02699050110103319.
61. Lee N, Fermo J. Warfarin and royal jelly interaction. Pharmacotherapy. 2006 Apr;26(4):583-6. doi: 10.1592/phco.26.4.583.

62. Khalid, Z.; Osuagwu, F.C.; Shah, B.; Roy, N.; Dillon, J.E.; Bradley, R. Celery root extract as an inducer of mania induction in a patient on venlafaxine and St John's Wort. *Postgrad. Med.* **2016**, *128*, 682–683, doi:10.1080/00325481.2016.1218263.
63. Al Faraj, S. Antagonism of the anticoagulant effect of warfarin caused by the use of *Commiphora molmol* as a herbal medication: A case report. *Ann. Trop. Med. Parasitol.* **2005**, *99*, 219–220, doi:10.1179/136485905x17434.
64. Ernst, E.; Pittler, M. Herbal medicine. *Med. Clin. North Am.* **2002**, *86*, 149–161, doi:10.1016/s0025-7125(03)00077-4.
65. Bruno, J.J.; Ellis, J. Herbal Use among US Elderly: 2002 National Health Interview Survey. *Ann. Pharmacother.* **2005**, *39*, 643–648, doi:10.1345/aph.1e460.
66. Kelly, J.P.; Kaufman, D.W.; Kelley, K.; Rosenberg, L.; Anderson, T.E.; Mitchell, A.A. Recent Trends in Use of Herbal and Other Natural Products. *Arch. Intern. Med.* **2005**, *165*, 281–286, doi:10.1001/archinte.165.3.281.
67. McHugh, M.L. Interrater reliability: The kappa statistic. *Biochem. Med.* **2012**, *22*, 276–282.
68. Ventola, C.L. Big data and pharmacovigilance: Data mining for adverse drug events and interactions. *Pharm. Ther.* **2018**, *43*, 340–351.
69. National Center for Biotechnology Information (NCBI). National Library of Medicine (US), National Center for Biotechnology Information: Bethesda, MD, USA. Available online: https://www.ncbi.nlm.nih.gov/ (accessed on May 18, 2021).
70. Elsevier. Embase®. Available online: https://www.elsevier.com/solutions/embase-biomedical-research (accessed on May 18, 2021).
71. IBM Micromedex. Truven Health Analytics/IBM Watson Health; 2020. Available online: https://www.micromedexsolutions.com (accessed on 18 May 2021).
72. Tatro, D. (Ed.) *Drug Interaction Facts: The Authority on Drug Interactions*; Wolters Kluwer Health. Philadelphia, PA, USA. 2015.
73. Walsh, K.M. Getting to Yes. J. Am. Geriatr. Soc. 2005, 53, 1072, doi:10.1111/j.1532-5415.2005.53326.x.
74. Mazaleuskaya, L.L.; Theken, K.; Gong, L.; Thorn, C.F.; Fitzgerald, G.A.; Altman, R.; Klein, T.E. PharmGKB summary: ibuprofen pathways. *Pharm. Genom.* **2015**, *25*, 96–106, doi:10.1097/fpc.0000000000000113.
75. Gurley, B.J.; Gardner, S.F.; Hubbard, M.A.; Williams, D.K.; Gentry, W.B.; Khan, I.A.; Shah, A. In Vivo Effects of Goldenseal, Kava Kava, Black Cohosh, and Valerian on Human Cytochrome P450 1A2, 2D6, 2E1, and 3A4 Phenotypes. *Clin. Pharmacol. Ther.* **2005**, *77*, 415–426.
76. Gurley, B.J.; Barone, G.W.; Williams, D.K.; Carrier, J.; Breen, P.; Yates, C.R.; Song, P.-F.; Hubbard, M.A.; Tong, Y.; Cheboyina, S. Effect of milk thistle (Silybum marianum) and black
77. Abdul, M.I.M.; Jiang, X.; Williams, K.M.; Day, R.; Roufogalis, B.; Liauw, W.S.; Xu, H.; McLachlan, A.J. Pharmacodynamic interaction of warfarin with cranberry but not with garlic in healthy subjects. *Br. J. Pharmacol.* **2008**, *154*, 1691–1700, doi:10.1038/bjp.2008.210.
78. Gurley, B.J.; Gardner, S.F.; Hubbard, M.A.; Williams, D.K.; Gentry, W.B.; Cui, Y.; Ang, C.Y. Cytochrome P450 phenotypic ratios for predicting herb-drug interactions in humans. *Clin. Pharmacol. Ther.* **2002**, *72*, 276–287, doi:10.1067/mcp.2002.126913.
79. Gurley, B.J.; Gardner, S.F.; Hubbard, M.A.; Williams, D.K.; Gentry, W.B.; Cui, Y.; Ang, C.Y. Clinical assessment of botanical supplementation on cytochrome P450 phenotypes in the elderly: St. John's wort, garlic oil, *Panax ginseng*, and *Ginkgo biloba*. *Drugs Aging* **2005**, *22*, 525–539.
80. Zhou, Q.; Ye, Z.; Ruan, Z.; Zeng, S. Investigation on modulation of human P-gp by multiple doses of Radix Astragali extract granules using fexofenadine as a phenotyping probe. *J. Ethnopharmacol.* **2013**, *146*, 744–749, doi:10.1016/j.jep.2013.01.037.
81. Fan, L.; Zhang, W.; Guo, D.; Tan, Z.-R.; Xu, P.; Li, Q.; Liu, Y.-Z.; Zhang, L.; He, T.-Y.; Hu, D.-L.; et al. The Effect of Herbal Medicine Baicalin on Pharmacokinetics of Rosuvastatin, Substrate of Organic Anion-transporting Polypeptide 1B1. *Clin. Pharmacol. Ther.* **2008**, *83*, 471–476, doi:10.1038/sj.clpt.6100318.
82. Hu, M.; Mak, V.W.L.; Yin, O.Q.P.; Chu, T.T.W.; Tomlinson, B. Effects of Grapefruit Juice and SLCO1B1 388A>G Polymorphism on the Pharmacokinetics of Pitavastatin. *Drug Metab. Pharmacokinet.* **2013**, *28*, 104–108, doi:10.2133/dmpk.dmpk-12-rg-067.
83. Nicandro, J.P.; Tsourounis, C.; Frassetto, L.; Guglielmo, B.J. In vivo effect of I'm-Yunity on hepatic cytochrome P450 3A4. *J. Herb. Pharmacother.* **2007**, *7*, 39–56.
84. Tankanow, R.; Tamer, H.R.; Streetman, D.S.; Smith, S.G.; Welton, J.L.; Annesley, T.; Aaronson, K.D.; Bleske, B.E. Interaction Study between Digoxin and a Preparation of Hawthorn (*Crataegus oxyacantha*). *J. Clin. Pharmacol.* **2003**, *43*, 637–642, doi:10.1177/0091270003253417.
85. Walker, A.F.; Marakis, G.; Simpson, E.; Hope, J.L.; Robinson, P.A.; Hassanein, M.; Simpson, H.C. Hypotensive effects of hawthorn for patients with diabetes taking prescription drugs: A randomized controlled trial. *Br. J. Gen. Pract.* **2006**, *56*, 437–443.

86. Ikehata, M.; Ohnishi, N.; Egami, S.; Kishi, H.; Shin, Y.; Takara, K.; Tsuchishita, Y.; Tokuda, N.; Hori, S.; Yatani, Y.; et al. Effects of Turmeric Extract on the Pharmacokinetics of Nifedipine After a Single Oral Administration in Healthy Volunteers. *J. Diet. Suppl.* **2008**, *5*, 401–410, doi:10.1080/19390210802519713.
87. Kim, T.-E.; Ha, N.; Kim, Y.; Kim, H.; Lee, J.W.; Jeon, J.-Y.; Kim, M.-G. Effect of epigallocatechin-3-gallate, major ingredient of green tea, on the pharmacokinetics of rosuvastatin in healthy volunteers. *Drug Des. Dev. Ther.* **2017**, *11*, 1409–1416, doi:10.2147/dddt.s130050.
88. Moltó, J.; Valle, M.; Miranda, C.; Cedeño, S.; Negredo, E.; Clotet, B. Herb-Drug Interaction between Echinacea purpurea and Etravirine in HIV-Infected Patients. *Antimicrob. Agents Chemother.* **2012**, *56*, 5328–5331, doi:10.1128/aac.01205-12.
89. Salman, S.A.B.; Amrah, S.; Wahab, M.S.A.; Ismail, Z.; Ismail, R.; Yuen, K.H.; Gan, S.H.; Msc, S.A.B.S.; Mpharm, R.I. Modification of propranolol's bioavailability by Eurycoma longifolia water-based extract. *J. Clin. Pharm. Ther.* **2010**, *35*, 691–696, doi:10.1111/j.1365-2710.2009.01147.x.
90. Xiao, C.-Q.; Chen, R.; Lin, J.; Wang, G.; Chen, Y.; Tan, Z.-R.; Zhou, H.-H. Effect of genistein on the activities of cytochrome P450 3A and P-glycoprotein in Chinese healthy participants. *Xenobiotica* **2011**, *42*, 173–178, doi:10.3109/00498254.2011.615954.
91. Aruna, D.; Naidu, M.U.R. Pharmacodynamic interaction studies of *Ginkgo biloba* with cilostazol and clopidogrel in healthy human subjects. *Br. J. Clin. Pharmacol.* **2007**, *63*, 333–338, doi:10.1111/j.1365-2125.2006.02759.x.
92. Dai, L.-L.; Fan, L.; Wu, H.-Z.; Tan, Z.-R.; Chen, Y.; Peng, X.-D.; Shen, M.-X.; Yang, G.-P.; Zhou, H.-H. Assessment of a pharmacokinetic and pharmacodynamic interaction between simvastatin and *Ginkgo biloba* extracts in healthy subjects. *Xenobiotica* **2013**, *43*, 862–867, doi:10.3109/00498254.2013.773385.
93. Fan, L.; Tao, G.-Y.; Wang, G.; Chen, Y.; Zhang, W.; He, Y.-J.; Li, Q.; Lei, H.-P.; Jiang, F.; Hu, D.-L.; et al. Effects of *Ginkgo biloba* Extract Ingestion on the Pharmacokinetics of Talinolol in Healthy Chinese Volunteers. *Ann. Pharmacother.* **2009**, *43*, 944–949, doi:10.1345/aph.1l656.
94. Guo, C.-X.; Pei, Q.; Yin, J.-Y.; Peng, X.; Zhou, B.-T.; Zhao, Y.-C.; Wu, L.-X.; Meng, X.-G.; Wang, G.; Li, Q.; et al. Effects of *Ginkgo biloba* extracts on pharmacokinetics and efficacy of atorvastatin based on plasma indices. *Xenobiotica* **2012**, *42*, 784–790, doi:10.3109/00498254.2012.661100.
95. Jiang, X.; Blair, E.Y.L.; McLachlan, A.J. Investigation of the Effects of Herbal Medicines on Warfarin Response in Healthy Subjects: A Population Pharmacokinetic-Pharmacodynamic Modeling Approach. *J. Clin. Pharmacol.* **2006**, *46*, 1370–1378, doi:10.1177/0091270006292124.
96. Kim, B.-H.; Kim, K.-P.; Lim, K.S.; Kim, J.-R.; Yoon, S.H.; Cho, J.-Y.; Lee, Y.-O.; Lee, K.-H.; Jang, I.-J.; Shin, S.-G.; et al. Influence of *Ginkgo biloba* extract on the pharmacodynamic effects and pharmacokinetic properties of ticlopidine: An open-label, randomized, two-period, two-treatment, two-sequence, single-dose crossover study in healthy Korean male volunteers. *Clin. Ther.* **2010**, *32*, 380–390, doi:10.1016/j.clinthera.2010.01.027.
97. Mohutsky, M.A.; Anderson, G.D.; Miller, J.W.; Elmer, G.W. *Ginkgo biloba*: Evaluation of CYP2C9 Drug Interactions In Vitro and In Vivo. *Am. J. Ther.* **2006**, *13*, 24–31, doi:10.1097/01.mjt.0000143695.68285.31.
98. Zadoyan, G.; Rokitta, D.; Klement, S.; Dienel, A.; Hoerr, R.; Gramatté, T.; Fuhr, U. Effect of *Ginkgo biloba* special extract EGb 761® on human cytochrome P450 activity: A cocktail interaction study in healthy volunteers. *Eur. J. Clin. Pharmacol.* **2012**, *68*, 553–560, doi:10.1007/s00228-011-1174-5.
99. Van Breemen, R.B.; Chen, L.; Tonsing-Carter, A.; Banuvar, S.; Barengolts, E.; Viana, M.; Chen, S.-N.; Pauli, G.F.; Bolton, J.L. Pharmacokinetic Interactions of a Hop Dietary Supplement with Drug Metabolism in Perimenopausal and Postmenopausal Women. *J. Agric. Food Chem.* **2020**, *68*, 5212–5220, doi:10.1021/acs.jafc.0c01077.
100. Gurley, B.J.; Swain, A.; Barone, G.W.; Williams, D.K.; Breen, P.; Yates, C.R.; Stuart, L.B.; Hubbard, M.A.; Tong, Y.; Cheboyina, S.; et al. Effect of Goldenseal (*Hydrastis canadensis*) and Kava Kava (*Piper methysticum*) Supplementation on Digoxin Pharmacokinetics in Humans. Drug Metab. Dispos. 2006, 35, 240–245, doi:10.1124/dmd.106.012708.
101. Gurley, B.J.; Swain, A.; Hubbard, M.A.; Hartsfield, F.; Thaden, J.; Williams, D.K.; Gentry, W.B.; Tong, Y. Supplementation With Goldenseal (*Hydrastis canadensis*), but not Kava Kava (*Piper methysticum*), Inhibits Human CYP3A Activity In Vivo. *Clin. Pharmacol. Ther.* **2008**, *83*, 61–69, doi:10.1038/sj.clpt.6100222.
102. Andrén, L.; Andreasson, A.; Eggertsen, R. Interaction between a commercially available St. John's wort product (Movina) and atorvastatin in patients with hypercholesterolemia. *Eur. J. Clin. Pharmacol.* **2007**, *63*, 913–916, doi:10.1007/s00228-007-0345-x.
103. Bell, E.C.; Ravis, W.R.; Chan, H.M.; Lin, Y.-J. Lack of Pharmacokinetic Interaction Between St. John's Wort and Prednisone. *Ann. Pharmacother.* **2007**, *41*, 1819–1824, doi:10.1345/aph.1k316.
104. Bell, E.C.; Ravis, W.R.; Lloyd, K.B.; Stokes, T.J. Effects of St. John's Wort Supplementation on Ibuprofen Pharmacokinetics. *Ann. Pharmacother.* **2007**, *41*, 229–234, doi:10.1345/aph.1h602.

105. Nieminen, T.H.; Hagelberg, N.M.; Saari, T.I.; Neuvonen, M.; Laine, K.; Neuvonen, P.; Olkkola, K.T. St John's wort greatly reduces the concentrations of oral oxycodone. *Eur. J. Pain* **2010**, *14*, 854–859, doi:10.1016/j.ejpain.2009.12.007.
106. Fan, L.; Zhou, G.; Guo, D.; Liu, Y.-L.; Chen, W.-Q.; Liu, Z.; Tan, Z.-R.; Sheng, D.; Zhou, H.-H.; Zhang, W. The Pregnane X Receptor Agonist St John's Wort Has No Effects on the Pharmacokinetics and Pharmacodynamics of Repaglinide. *Clin. Pharmacokinet.* **2011**, *50*, 605–611, doi:10.2165/11587310-000000000-00000.
107. Goey, A.K.L.; Meijerman, I.; Rosing, H.; Marchetti, S.; Mergui-Roelvink, M.; Keessen, M.; Burgers, J.A.; Beijnen, J.H.; Schellens, J.H.M. The Effect of St John's Wort on the Pharmacokinetics of Docetaxel. Clin. Pharmacokinet. 2013, 53, 103–110, doi:10.1007/s40262-013-0102-5.
108. Hennessy, M.; Kelleher, D.; Spiers, P.; Barry, M.; Kavanagh, P.; Back, D.; Mulcahy, F.; Feely, J. St John's Wort increases expression of P-glycoprotein: Implications for drug interactions. *Br. J. Clin. Pharmacol.* **2002**, *53*, 75–82, doi:10.1046/j.0306-5251.2001.01516.x.
109. Jiang, X.; Williams, K.M.; Liauw, W.S.; Ammit, A.; Roufogalis, B.; Duke, C.C.; Day, R.; McLachlan, A.J. Effect of St John's wort and ginseng on the pharmacokinetics and pharmacodynamics of warfarin in healthy subjects. *Br. J. Clin. Pharmacol.* **2004**, *57*, 592–599, doi:10.1111/j.1365-2125.2003.02051.x.
110. Loughren, M.J.; Kharasch, E.D.; Kelton-Rehkopf, M.C.; Syrjala, K.L.; Shen, D.D. Influence of St. John's Wort on Intravenous Fentanyl Pharmacokinetics, Pharmacodynamics, and Clinical Effects. *Anesthesiology* **2020**, *132*, 491–503, doi:10.1097/aln.0000000000003065.
111. Markert, C.; Kastner, I.M.; Hellwig, R.; Kalafut, P.; Schweizer, Y.; Hoffmann, M.M.; Burhenne, J.; Weiss, J.; Mikus, G.; Haefeli, W.E. The Effect of Induction of CYP3A4 by St John's Wort on Ambrisentan Plasma Pharmacokinetics in Volunteers of knownCYP2C19Genotype. *Basic Clin. Pharmacol. Toxicol.* **2014**, *116*, 423–428, doi:10.1111/bcpt.12332.
112. Mueller, S.C.; Majcher-Peszynska, J.; Uehleke, B.; Klammt, S.; Mundkowski, R.G.; Miekisch, W.; Sievers, H.; Bauer, S.; Frank, B.; Kundt, G.; et al. The extent of induction of CYP3A by St. John's wort varies among products and is linked to hyperforin dose. *Eur. J. Clin. Pharmacol.* **2006**, *62*, 29–36, doi:10.1007/s00228-005-0061-3.
113. Mueller, S.C.; Majcher-Peszynska, J.; Mundkowski, R.G.; Uehleke, B.; Klammt, S.; Sievers, H.; Lehnfeld, R.; Frank, B.; Thurow, K.; Kundt, G.; et al. No clinically relevant CYP3A induction after St. John's wort with low hyperforin content in healthy volunteers. *Eur. J. Clin. Pharmacol.* **2008**, *65*, 81–87, doi:10.1007/s00228-008-0554-y.
114. Murphy, P.A.; Kern, S.E.; Stanczyk, F.Z.; Westhoff, C.L. Interaction of St. John's Wort with oral contraceptives: Effects on the pharmacokinetics of norethindrone and ethinyl estradiol, ovarian activity and breakthrough bleeding. *Contracept.* **2005**, *71*, 402–408, doi:10.1016/j.contraception.2004.11.004.
115. Piscitelli, S.C.; Burstein, A.H.; Chaitt, D.; Alfaro, R.M.; Falloon, J. Indinavir concentrations and St John's wort. *Lancet* **2000**, *355*, 547–548, doi:10.1016/s0140-6736(99)05712-8.
116. Portolés, A.; Terleira, A.; Calvo, A.; Martínez, M.I.; Resplandy, G. Effects of *Hypericum perforatum* on Ivabradine Pharmacokinetics in Healthy Volunteers: An Open-Label, Pharmacokinetic Interaction Clinical Trial. *J. Clin. Pharmacol.* **2006**, *46*, 1188–1194, doi:10.1177/0091270006291623.
117. Xie, R.; Tan, L.H.; Polasek, E.C.; Hong, C.; Teillol-Foo, M.; Gordi, T.; Sharma, A.; Ms, D.J.N.; Arakawa, T.; Knuth, D.W.; et al. CYP3A and P-Glycoprotein Activity Induction With St. John's Wort in Healthy Volunteers From 6 Ethnic Populations. *J. Clin. Pharmacol.* **2005**, *45*, 352–356, doi:10.1177/0091270004273320.
118. Xu, H.; Williams, K.M.; Liauw, W.S.; Murray, M.; Day, R.; McLachlan, A.J. Effects of St John's wort and CYP2C9 genotype on the pharmacokinetics and pharmacodynamics of gliclazide. *Br. J. Pharmacol.* **2008**, *153*, 1579–1586, doi:10.1038/sj.bjp.0707685.
119. Heger-Mahn, D.; Pabst, G.; Dienel, A.; Schlafke, S.; Klipping, C. No interacting influence of lavender oil preparation silexan on oral contraception using an ethinyl estradiol/levonorgestrel combination. *Drugs R&D* **2014**, *14*, 265–272, doi:10.1007/s40268-014-0065-5.
120. Chen, Y.; Ouyang, D.-S.; Kang, Z.; Yang, G.-P.; Tan, Z.-R.; Zhou, G.; Yan, J. Effect of a traditional Chinese medicine Liu Wei Di Huang Wan on the activities of CYP2C19, CYP2D6 and CYP3A4 in healthy volunteers. *Xenobiotica* **2011**, *42*, 596–602, doi:10.3109/00498254.2011.644596.
121. Fan, L.; Wang, G.; Wang, L.-S.; Chen, Y.; Zhang, W.; Huang, Y.-F.; Huang, R.-X.; Hu, D.-L.; Wang, D.; Zhou, H.-H. Herbal medicine Yin Zhi Huang induces CYP3A4-mediated sulfoxidation and CYP2C19-dependent hydroxylation of omeprazole. *Acta Pharmacol. Sin.* **2007**, *28*, 1685–1692, doi:10.1111/j.1745-7254.2007.00617.x.
122. Kim, H.; Bae, S.K.; Park, S.-J.; Shim, E.-J.; Kim, H.-S.; Shon, J.-H.; Liu, K.-H.; Shin, J.-G. Effects of woohwangcheongsimwon suspension on the pharmacokinetics of bupropion and its active metabolite, 4-hydroxybupropion, in healthy subjects. *Br. J. Clin. Pharmacol.* **2010**, *70*, 126–131, doi:10.1111/j.1365-2125.2010.03661.x.

123. Nakao, M.; Muramoto, Y.; Hisadome, M.; Yamano, N.; Shoji, M.; Fukushima, Y.; Saruwatari, J.; Nakagawa, K. The effect of Shoseiryuto, a traditional Japanese medicine, on cytochrome P450s, N-acetyltransferase 2 and xanthine oxidase, in extensive or intermediate metabolizers of CYP2D6. *Eur. J. Clin. Pharmacol.* **2007**, *63*, 345–353, doi:10.1007/s00228-006-0253-5.
124. Park, S.-I.; Park, J.-Y.; Park, M.-J.; Yim, S.-V.; Kim, B.-H. Effects of Ojeok-san on the Pharmacokinetics of Celecoxib at Steady-state in Healthy Volunteers. *Basic Clin. Pharmacol. Toxicol.* **2018**, *123*, 51–57, doi:10.1111/bcpt.12971.
125. Saruwatari, J.; Takaishi, C.; Yoshida, K.; Takashima, A.; Fujimura, Y.; Umemoto, Y.; Abe, T.; Kitamado, M.; Shimomasuda, M.; Muramoto, Y.; et al. A herbal-drug interaction study of keishi-bukuryo-gan, a traditional herbal preparation used for menopausal symptoms, in healthy female volunteers. *J. Pharm. Pharmacol.* **2012**, *64*, 670–676, doi:10.1111/j.2042-7158.2011.01443.x.
126. Wang, P.; Sun, H.; Yang, L.; Li, L.-Y.; Hao, J.; Ruff, D.; Guo, Z.-X. Absence of an effect of T89 on the steady-state pharmacokinetics and pharmacodynamics of warfarin in healthy volunteers. *J. Clin. Pharmacol.* **2014**, *54*, 234–239, doi:10.1002/jcph.209.
127. Lee, Y.H.; Lee, B.K.; Choi, Y.J.; Yoon, I.K.; Chang, B.C.; Gwak, H.S. Interaction between warfarin and Korean red ginseng in patients with cardiac valve replacement. *Int. J. Cardiol.* **2010**, *145*, 275–276, doi:10.1016/j.ijcard.2009.09.553.
128. Malati, C.Y.; Robertson, S.M.; Hunt, J.D.; Chairez, C.; Alfaro, R.M.; Kovacs, J.A.; Penzak, S.R. Influence of *Panax ginseng* on Cytochrome P450 (CYP)3A and P-glycoprotein (P-gp) Activity in Healthy Participants. *J. Clin. Pharmacol.* **2012**, *52*, 932–939, doi:10.1177/0091270011407194.
129. Fatima, N.; Pingali, U.; Muralidhar, N. Study of pharmacodynamic interaction of *Phyllanthus emblica* extract with clopidogrel and ecosprin in patients with type II diabetes mellitus. *Phytomedicine* **2014**, *21*, 579–585, doi:10.1016/j.phymed.2013.10.024.
130. Bedada, S.K.; Neerati, P. Resveratrol Pretreatment Affects CYP2E1 Activity of Chlorzoxazone in Healthy Human Volunteers. *Phytotherapy Res.* **2016**, *30*, 463–468, doi:10.1002/ptr.5549.
131. Fuhr, U.; Beckmann-Knopp, S.; Jetter, A.; Lück, H.; Mengs, U. The Effect of Silymarin on Oral Nifedipine Pharmacokinetics. *Planta Medica* **2007**, *73*, 1429–1435, doi:10.1055/s-2007-990256.
132. Kawaguchi-Suzuki, M.; Frye, R.F.; Zhu, H.-J.; Brinda, B.J.; Chavin, K.D.; Bernstein, H.J.; Markowitz, J.S. The Effects of Milk Thistle (*Silybum marianum*) on Human Cytochrome P450 Activity. *Drug Metab. Dispos.* **2014**, *42*, 1611–1616, doi:10.1124/dmd.114.057232.
133. Tocaciu, S.; Oliver, L.J.; Lowenthal, R.M.; Peterson, G.M.; Patel, R.; Shastri, M.; McGuinness, G.; Olesen, I.; Fitton, J.H. The Effect of *Undaria pinnatifida* Fucoidan on the Pharmacokinetics of Letrozole and Tamoxifen in Patients With Breast Cancer. *Integr. Cancer Ther.* **2018**, *17*, 99–105, doi:10.1177/1534735416684014.
134. Thronicke, A.; Steele, M.L.; Grah, C.; Matthes, B.; Schad, F. Clinical safety of combined therapy of immune checkpoint inhibitors and Viscum album L. therapy in patients with advanced or metastatic cancer. *BMC Complement. Altern. Med.* **2017**, *17*, 534, doi:10.1186/s12906-017-2045-0.
135. Açikgöz, S.K.; Açıkgöz, E. Gastrointestinal bleeding secondary to interaction of Artemisia absinthium with warfarin. *Drug Metab. Drug Interact.* **2013**, *28*, 187–189, doi:10.1515/dmdi-2013-0021.
136. Almeida, J.; Grimsley, E. Coma from the Health Food Store: Interaction between Kava and Alprazolam. *Ann. Intern. Med.* **1996**, *125*, 940–941.
137. Alscher, D.M.; Klotz, U. Drug interaction of herbal tea containing St. John's wort with cyclosporine. *Transpl. Int.* **2003**, *16*, 543–544, doi:10.1111/j.1432-2277.2003.tb00345.x.
138. Aslam, M. Stockley, I. Interaction between curry ingredient (karela) and drug (chlorpropamide). *Lancet* **1979**, *313*, 607, doi:10.1016/s0140-6736(79)91028-6.
139. Barbenel, D.M.; Yusufi, B.; Bench, C.J.; O'Shea, D. Mania in a patient receiving testosterone replacement post-orchidectomy taking St John's wort and sertraline. *J. Psychopharmacol.* **2000**, *14*, 84–86, doi:10.1177/026988110001400113.
140. Barone, G.W.; Gurley, B.J.; Ketel, B.L.; Lightfoot, M.L.; Abul-Ezz, S.R. Drug Interaction between St. John's Wort and Cyclosporine. *Ann. Pharmacother.* **2000**, *34*, 1013–1016, doi:10.1345/aph.10088.
141. Bilgi, N.; Bell, K.; Ananthakrishnan, A.N.; Atallah, E. Imatinib and *Panax ginseng*: A Potential Interaction Resulting in Liver Toxicity. *Ann. Pharmacother.* **2010**, *44*, 926–928, doi:10.1345/aph.1m715.
142. Bolley, R.; Zülke, C.; Kammerl, M.; Fischereder, M.; Krämer, B.K. Tacrolimus-induced nephrotoxicity unmasked by induction of the CYP3A4 system with ST JOHN'S wort. *Transplantation* **2002**, *73*, 1009, doi:10.1097/00007890-200203270-00035.

143. Bossaer, J.B.; Odle, B.L. Probable Etoposide Interaction with Echinacea. *J. Diet. Suppl.* **2012**, *9*, 90–95, doi:10.3109/19390211.2012.682643.
144. Breidenbach, T.; Hoffmann, M.W.; Becke, T.; Schlitt, H.; Klempnauer, J. Drug interaction of St John's wort with ciclosporin. *Lancet* **2000**, *355*, 1912.
145. Buckley, M.S.; Goff, A.D.; Knapp, W.E. Fish Oil Interaction with Warfarin. *Ann. Pharmacother.* **2004**, *38*, 50–53, doi:10.1345/aph.1d007.
146. Bamgbade, O. The perioperative implications of khat use. *Eur. J. Anaesthesiol.* **2008**, *25*, 170–172, doi:10.1017/s026502150700258x.
147. Campos, M.; Machado, J.; Costa, M.; Lino, S.; Correia, F.; Maltez, F. Case Report: Severe Hematological, Muscle and Liver Toxicity Caused by Drugs and Artichoke Infusion Interaction in an Elderly Polymedicated Patient. *Curr. Drug Saf.* **2018**, *13*, 44–50, doi:10.2174/1574886312666170912163746.
148. Cappuzzo, K.A. Herbal Product Use in a Patient with Polypharmacy. *Consult. Pharm.* **2006**, *21*, 911–915, doi:10.4140/tcp.n.2006.911.
149. Carbajal, R.; Yisfalem, A.; Pradhan, N.; Baumstein, D.; Chaudhari, A. Case Report: Boldo (*Peumus boldus*) and Tacrolimus Interaction in a Renal Transplant Patient. *Transplant. Proc.* **2014**, *46*, 2400–2402, doi:10.1016/j.transproceed.2014.01.021.
150. Carr, M.; Klotz, J.; Bergeron, M. Coumadin resistance and the vitamin supplement "noni". *Am. J. Hematol.* **2004**, *77*, 103, doi:10.1002/ajh.20135.
151. Carrasco, M.C.; Vallejo, J.R.; Pardo-de-Santayana, M.; Peral, D.; Martín, M.Á.; Altimiras, J. Interactions of *Valeriana officinalis* L. and *Passiflora incarnata* L. in a patient treated with lorazepam. *Phytother. Res.* **2009**, *23*, 1795–1796, doi:10.1002/ptr.2847.
152. Carter, J.; Yeh, R.F.; Braunschweig, I.; Barta, S. Unreported use of an herbal supplement resulting in decreased clearance of intravenous busulfan in a patient undergoing auto-SCT. *Bone Marrow Transplant.* **2014**, *49*, 313–314, doi:10.1038/bmt.2013.169.
153. Cattaneo, D.; Fusi, M.; Gervasoni, C. No effects of Hypericum-containing complex on dolutegravir plasma trough concentrations: A case report. *Eur. J. Clin. Pharmacol.* **2019**, *75*, 1467–1468, doi:10.1007/s00228-019-02714-0.
154. Chan, J.C.M.; Ng, M.-H.; Wong, R.S.M.; Tomlinson, B. A case of simvastatin-induced myopathy with SLCO1B1 genetic predisposition and co-ingestion of linagliptin and *Stevia rebaudiana*. *J. Clin. Pharm. Ther.* **2019**, *44*, 381–383, doi:10.1111/jcpt.12805.
155. Chang, Y.-Y.; Liu, J.-S.; Lai, S.-L.; Wu, H.-S.; Lan, M.-Y. Cerebellar Hemorrhage Provoked by Combined Use of Nattokinase and Aspirin in a Patient with Cerebral Microbleeds. *Intern. Med.* **2008**, *47*, 467–469, doi:10.2169/internalmedicine.47.0620.
156. Constable, S.; Ham, A.; Pirmohamed, M. Herbal medicines and acute medical emergency admissions to hospital. *Br. J. Clin. Pharmacol.* **2007**, *63*, 247–248, doi:10.1111/j.1365-2125.2006.02817.x.
157. Cordova, E.; Morganti, L.; Rodriguez, C. Possible Drug–Herb Interaction between Herbal Supplement Containing Horsetail (*Equisetum arvense*) and Antiretroviral Drugs. *J. Int. Assoc. Provid. AIDS Care (JIAPAC)* **2017**, *16*, 11–13, doi:10.1177/2325957416680295.
158. Damato, A.; Larocca, M.; Rondini, E.; Menga, M.; Pinto, C.; Versari, A. Severe Rhabdomyolysis during Treatment with Trabectedin in Combination with a Herbal Drug in a Patient with Metastatic Synovial Sarcoma: A Case Report. *Case Reports in Oncology* **2017**, *10*, 258–264, doi:10.1159/000464440.
159. Dannawi, M. Possible serotonin syndrome after combination of buspirone and St John's Wort. *J. Psychopharmacol.* **2002**, *16*, 401, doi:10.1177/026988110201600420.
160. Daveluy, A.; Géniaux, H.; Thibaud, L.; Mallaret, M.; Miremont-Salamé, G.; Haramburu, F. Probable Interaction Between an Oral Vitamin K Antagonist and Turmeric (*Curcuma longa*). *Therapie* **2014**, *69*, 519–520, doi:10.2515/therapie/2014062.
161. Epstein, R.J.; Leung, T.W.T.; Cheung, P.S.Y. Panmucositis and chemosensitisation associated with betel quid chewing during dose-dense adjuvant breast cancer chemotherapy. *Cancer Chemother. Pharmacol.* **2006**, *58*, 835–837, doi:10.1007/s00280-006-0218-5.
162. Galera, R.M.L.; Pascuet, E.R.; Mur, J.I.E.; Montoro-Ronsano, J.B.; Juarez-Gimenez, J.C. Interaction between cat's claw and protease inhibitors atazanavir, ritonavir and saquinavir. *Eur. J. Clin. Pharmacol.* **2008**, *64*, 1235–1236, doi:10.1007/s00228-008-0551-1.
163. Galluzzi, S.; Zanetti, O.; Binetti, G.; Trabucchi, M.; Frisoni, G.B. Coma in a patient with Alzheimer's disease taking low dose trazodone and *Ginkgo biloba*. *J. Neurol. Neurosurg. Psychiatry* **2000**, *68*, 679–680, doi:10.1136/jnnp.68.5.679a.
164. Hamann, G.L.; Campbell, J.D.; George, C.M. Warfarin-Cranberry Juice Interaction. *Ann. Pharmacother.* **2011**, *45*, 420, doi:10.1345/aph.1p451.
165. Gordon, R.Y.; Becker, D.J.; Rader, D.J. Reduced Efficacy of Rosuvastatin by St. John's Wort. *Am. J. Med.* **2009**, *122*, e1–e2, doi:10.1016/j.amjmed.2008.09.033.
166. Griffiths, A.; Beddall, A.; Pegler, S. Fatal haemopericardium and gastrointestinal haemorrhage due to possible interaction of cranberry juice with warfarin. *J. R. Soc. Promot. Health* **2008**, *128*, 324–326, doi:10.1177/1466424008096615.
167. Hanselin, M. INR elevation with maitake extract in combination with warfarin. *Ann. Pharmacother.* **2010**, *44*, 223–224, doi:10.1345/aph.1m052.

168. Hou, Q.; Han, W.; Fu, X. Pharmacokinetic interaction between tacrolimus and berberine in a child with idiopathic nephrotic syndrome. *Eur. J. Clin. Pharmacol.* **2013**, *69*, 1861–1862, doi:10.1007/s00228-013-1537-1.
169. Hughes, R.L. Fatal combination of mitragynine and quetiapine—A case report with discussion of a potential herb-drug interaction. *Forensic Sci. Med. Pathol.* **2018**, *15*, 110–113, doi:10.1007/s12024-018-0049-9.
170. Hurren, K.M.; Lewis, C.L. Probable interaction between warfarin and bee pollen. *Am. J. Health Pharm.* **2010**, *67*, 2034–2037, doi:10.2146/ajhp090489.
171. Hwang, S.-W.; Han, H.-S.; Lim, K.Y.; Han, J.-Y. Drug Interaction Between Complementary Herbal Medicines and Gefitinib. *J. Thorac. Oncol.* **2008**, *3*, 942–943, doi:10.1097/jto.0b013e3181803f1e.
172. Iida, R.; Otsuka, Y.; Matsumoto, K.; Kuriyama, S.; Hosoya, T. Pseudoaldosteronism due to the concurrent use of two herbal medicines containing glycyrrhizin: Interaction of glycyrrhizin with angiotensin-converting enzyme inhibitor. *Clin. Exp. Nephrol.* **2006**, *10*, 131–135, doi:10.1007/s10157-006-0415-x.
173. Janetzky, K.; Morreale, A.P. Drug Experience Warfarin, and ginseng. *Am. J. Healh Pharm.* **1997**, *54*, 692–693.
174. Ji, H.; Zhang, G.; Yue, F.; Zhou, X. Adverse event due to a likely interaction between sodium aescinate and *Ginkgo biloba* extract: A case report. *J. Clin. Pharm. Ther.* **2017**, *42*, 237–238, doi:10.1111/jcpt.12500.
175. Jones, B.D.; Runikis, A.M. Interaction of Ginseng with Phenelzine. *J. Clin. Psychopharmacol.* **1987**, *7*, 201–202, doi:10.1097/00004714-198706000-00030.
176. Kang, Y.-C.; Chen, M.-H.; Lai, S.-L. Potentially Unsafe Herb-drug Interactions Between a Commercial Product of Noni Juice and Phenytoin- A Case Report. *Acta Neurol. Taiwanica* **2015**, *24*, 43–46.
177. Karhova, M.; Trelchel, U.; Malagb, M.; Fnllmg, A.; Gerken, G.; Broelsch, C.E. Interaction of *Hypericum perforatum* (St. John's wort) with cycbsporin A metabolism in a patient after liver transplantation. *J. Hepatol.* **2000**, *33*, 853.
178. Cambria-Kiely, J.A. Effect of Soy Milk on Warfarin Efficacy. *Ann. Pharmacother.* **2002**, *36*, 1893–1896, doi:10.1345/aph.1c160.
179. Krüth, P.; Brosi, E.; Fux, R.; Mörike, K.; Gleiter, C.H. Ginger-Associated Overanticoagulation by Phenprocoumon. *Ann. Pharmacother.* **2004**, *38*, 257–260, doi:10.1345/aph.1d225.
180. Lam, A.Y.; Elmer, G.W.; Mohutsky, M.A. Possible interaction between warfarin and *Lycium barbarum* L. *Ann. Pharmacother.* **2001**, *35*, 1199–1201.
181. Lambert, J.-P.; Cormier, J. Potential Interaction between Warfarin and Boldo-Fenugreek. *Pharmacother. J. Hum. Pharmacol. Drug Ther.* **2001**, *21*, 509–512, doi:10.1592/phco.21.5.509.34492.
182. Lantz, M.S.; Buchalter, E.; Giambanco, V. St. John's wort and antidepressant drug interactions in the elderly. *J. Geriatr. Psychiatry Neurol.* **1999**, *12*, 7–10, doi:10.1177/089198879901200103.
183. Leung, H.; Hung, A.; Hui, A.; Chan, T. Warfarin overdose due to the possible effects of *Lycium barbarum* L. *Food Chem. Toxicol.* **2008**, *46*, 1860–1862, doi:10.1016/j.fct.2008.01.008.
184. Mai, I.; Krüger, H.; Budde, K.; Johne, A.; Brockmöller, J.; Neumayer, H.-H.; Roots, I. Hazardous pharmacokinetic interaction of Saint John's wort (*Hypericum perforatum*) with the immunosuppressant cyclosporin. *Int. J. Clin. Pharmacol. Ther.* **2000**, *38*, 500–502, doi:10.5414/cpp38500.
185. Mateo-Carrasco, H.; Gálvez-Contreras, M.C.; Fernández-Ginés, F.D.; Nguyen, T.V. Elevated liver enzymes resulting from an interaction between Raltegravir and *Panax ginseng*: A case report and brief review. *Drug Metab. Drug Interact.* **2012**, *27*, 171–175, doi:10.1515/dmdi-2012-0019.
186. McGovern, E.; McDonnell, T. Herbal Medicine—Sets the Heart Racing! *Ir. Med. J.* **2009**, *1*, 517–538.
187. McCrae, S. Elevated serum digoxin levels in a patient taking digoxin and Siberian ginseng. *CMAJ* **1996**, *155*, 293–295.
188. Meisel, C. Fatal intracerebral mass bleeding associated with *Ginkgo biloba* and ibuprofen. *Atherosclerosis* **2003**, *167*, 367, doi:10.1016/s0021-9150(03)00015-7.
189. Mendoza, C.E.; Ferreira, A.C.; De Marchena, E. Warfarin and herbal products interaction causing prosthetic aortic valve thrombosis presenting as acute myocardial infarction. *J. Hear. Valve Dis.* **2004**, *13*, 22–24.
190. Myers, A.P.; Watson, T.A.; Strock, S.B. Drug Reaction with Eosinophilia and Systemic Symptoms Syndrome Probably Induced by a Lamotrigine-Ginseng Drug Interaction. *Pharmacother. J. Hum. Pharmacol. Drug Ther.* **2015**, *35*, e9–e12, doi:10.1002/phar.1550.
191. Naccarato, M.; Yoong, D.; Gough, K. A Potential Drug–Herbal Interaction between *Ginkgo biloba* and Efavirenz. *J. Int. Assoc. Physicians AIDS Care* **2012**, *11*, 98–100, doi:10.1177/1545109711435364.

192. Nayeri, A.; Wu, S.; Adams, E.; Tanner, C.; Meshman, J.; Saini, I.; Reid, W. Acute Calcineurin Inhibitor Nephrotoxicity Secondary to Turmeric Intake: A Case Report. *Transplant. Proc.* **2017**, *49*, 198–200, doi:10.1016/j.transproceed.2016.11.029.
193. Nebel, A.; Schneider, B.J.; Baker, R.K.; Kroll, D.J. Potential metabolic interaction between St. John's wort and theophylline. *Ann. Pharmacother.* **1999**, *33*, 502.
194. Nowack, R.; Nowak, B. Herbal teas interfere with cyclosporin levels in renal transplant patients. *Nephrol. Dial. Transplant.* **2005**, *20*, 2554–2556, doi:10.1093/ndt/gfi003.
195. Orr, A.; Parker, R. Red clover causing symptoms suggestive of methotrexate toxicity in a patient on high-dose methotrexate. *Menopause Int. Integr. J. Postreprod. Health* **2013**, *19*, 133–134, doi:10.1177/1754045313502473.
196. Paeng, C.H.; Sprague, M.; Jackevicius, C.A. Interaction between Warfarin and Cranberry Juice. *Clin. Ther.* **2007**, *29*, 1730–1735, doi:10.1016/j.clinthera.2007.08.018.
197. Page, R.L.; Lawrence, J.D. Potentiation of Warfarin by Dong Quai. *Pharmacother. J. Hum. Pharmacol. Drug Ther.* **1999**, *19*, 870–876, doi:10.1592/phco.19.10.870.31558.
198. Jacquin-Porretaz, C.; Nardin, C.; Blanc, D.; Aubin, F.; Gérard, B.; Drobacheff-Thiebaut, C.; Jacoulet, P.; Westeel, V. Cutaneous Toxicity Induced by Hibiscus Tea in a Patient Treated with Erlotinib. *J. Thorac. Oncol.* **2017**, *12*, e47–e48, doi:10.1016/j.jtho.2017.01.010.
199. Prasad, K.; Tharangani, P.; Samaranayake, C. Recurrent relapses of depression in a patient established on sertraline after taking herbal medicinal mixtures—A herb–drug interaction? *J. Psychopharmacol.* **2008**, *23*, 216–219, doi:10.1177/0269881108089808.
200. Rindone, J.P.; Murphy, T.W. Warfarin-cranberry juice 9nteraction resulting in profound hypoprothrombinemia and bleeding. *Am. J. Ther.* **2005**, *13*, 283–284.
201. Rivera, C.A.; Ferro, C.L.; Bursua, A.J.; Gerber, B.S. Probable Interaction Between Lycium barbarum (Goji) and Warfarin. *Pharmacother. J. Hum. Pharmacol. Drug Ther.* **2012**, *32*, e50–e53, doi:10.1002/j.1875-9114.2012.01018.x.
202. Rosado, M.F. Thrombosis of a Prosthetic Aortic Valve Disclosing a Hazardous Interaction between Warfarin and a Commercial Ginseng Product. *Cardiol.* **2003**, *99*, 111, doi:10.1159/000069720.
203. Rozenfeld, V.; Crain, J.L.; Callahan, A.K. Possible augmentation of warfarin effect by glucosamine-chondroitin. *Am. J. Health Pharm.* **2004**, *61*, 306–307, doi:10.1093/ajhp/61.3.306.
204. Rubin, D.; McGovern, B.; Kopelman, R.I. Back to Basics. *Am. J. Med.* **2006**, *119*, 482–483, doi:10.1016/j.amjmed.2006.04.007.
205. Ruschitzka, F.; Meier, P.J.; Turina, M.; Lüscher, T.F.; Noll, G. Acute heart transplant rejection due to Saint John's wort. *Lancet* **2000**, *355*, 548–549, doi:10.1016/s0140-6736(99)05467-7.
206. Schwarz, U.I.; Büschel, B.; Kirch, W. Unwanted pregnancy on self-medication with St John's wort despite hormonal contraception. *Br. J. Clin. Pharmacol.* **2003**, *55*, 112–113, doi:10.1046/j.1365-2125.2003.01716.x.
207. Segal, R. Warfarin interaction with *Matricaria chamomilla*. *Can. Med. Assoc. J.* **2006**, *174*, 1281–1282, doi:10.1503/cmaj.051191.
208. Strippoli, S.; Lorusso, V.; Albano, A.; Guida, M. Herbal-drug interaction induced rhabdomyolysis in a liposarcoma patient receiving trabectedin. *BMC Complement. Altern. Med.* **2013**, *13*, 199, doi:10.1186/1472-6882-13-199.
209. Suvarna, R.; Pirmohamed, M.; Henderson, L. Possible interaction between warfarin and cranberry juice. *BMJ* **2003**, *327*, 1454, doi:10.1136/bmj.327.7429.1454.
210. Tam, L.S.; Chan, T.Y.K.; Leung, W.K.; Critchley, J.A.J.H. Warfarin interactions with Chinese traditional medicines: Danshen and methyl salicylate medicated oil. *Aust. N. Z. J. Med.* **1995**, *25*, 258, doi:10.1111/j.1445-5994.1995.tb01540.x.
211. Tamura, S.; Warabi, Y.; Matsubara, S. Severe liver dysfunction possibly caused by the combination of interferon beta-1b therapy and melilot (sweet clover) supplement. *J. Clin. Pharm. Ther.* **2012**, *37*, 724–725, doi:10.1111/j.1365-2710.2012.01350.x.
212. Taylor, J.; Wilt, V. Probably antagonism of warfarin by green tea. *Ann. Pharmacother.* **1999**, *33*, 426–428.
213. van den Bout, C.J.; Bosch, M.E.; Burger, D.M.; Koopmans, P.P.; van der Ven, A.J. Toxic lopinavir concentrations in an HIV-1 infected patient taking herbal medications. *AIDS* **2008**, *22*, 1243–1244, doi:10.1097/qad.0b013e32830261f4.
214. Van Strater, A.C.; Bogers, J.P. Interaction of St John's wort (*Hypericum perforatum*) with clozapine. *Int. Clin. Psychopharmacol.* **2012**, *27*, 121–124, doi:10.1097/yic.0b013e32834e8afd.
215. Welch, J.; Forster, K. Probably elevation in international normalized ratio from cranberry juice. *J. Pharm. Technol.* **2007**, *23*, 104–107.

216. Werba, J.P.; Misaka, S.; Giroli, M.G.; Shimomura, K.; Amato, M.; Simonelli, N.; Vigo, L.; Tremoli, E. Update of green tea interactions with cardiovascular drugs and putative mechanisms. *J. Food Drug Anal.* **2018**, *26*, S72–S77, doi:10.1016/j.jfda.2018.01.008.
217. Wiegman, D.-J.; Brinkman, K.; Franssen, E.J.F. Interaction of *Ginkgo biloba* with efavirenz. *AIDS* **2009**, *23*, 1184–1185, doi:10.1097/qad.0b013e32832c412b.
218. Wong, A.L.N.; Chan, T.Y.K. Interaction between warfarin and the herbal product quilinggao. *Ann. Pharmacother.* **2003**, *37*, 836–838, doi:10.1345/aph.1c503.
219. Yu, C.M.; Chan, J.; Sanderson, J.E. Chinese herbs and warfarin potentiation by 'Danshen'. *J. Intern. Med.* **1997**, *241*, 337–339, doi:10.1046/j.1365-2796.1997.134137000.x.
220. Kim, H.-S.; Kim, G.-Y.; Yeo, C.-W.; Oh, M.; Ghim, J.-L.; Shon, J.-H.; Kim, E.-Y.; Kim, D.H.; Shin, J.-G. The effect of *Ginkgo biloba* extracts on the pharmacokinetics and pharmacodynamics of cilostazol and its active metabolites in healthy Korean subjects. *Br. J. Clin. Pharmacol.* **2014**, *77*, 821–830, doi:10.1111/bcp.12236.
